# Diagnostic and Microbiological Impact of Multiplex Syndromic Testing for Acute Infectious Gastroenteritis in a Regional Laboratory Network: A Real-World Before–After Study

**DOI:** 10.3390/microorganisms14071559

**Published:** 2026-07-16

**Authors:** Massimiliano Guerra, Martina Brandolini, Laura Dionisi, Alessandra Mistral De Pascali, Ludovica Ingletto, Claudia Colosimo, Giulia Gatti, Maria Sofia Montanari, Anna Marzucco, Laura Grumiro, Giorgio Dirani, Silvia Zannoli, Alessandra Scagliarini, Vittorio Sambri, Monica Cricca

**Affiliations:** 1 Unit of Microbiology, The Greater Romagna Area Hub Laboratory, 47522 Cesena, Italy; martina.brandolini3@unibo.it (M.B.); giulia.gatti@auslromagna.it (G.G.); anna.marzucco@auslromagna.it (A.M.); giorgio.dirani@auslromagna.it (G.D.); silvia.zannoli@auslromagna.it (S.Z.); vittorio.sambri@unibo.it (V.S.); monica.cricca3@unibo.it (M.C.); 2 Department of Medical and Surgical Sciences (DIMEC), University of Bologna, 40138 Bologna, Italy; laura.dionisi2@unibo.it (L.D.); alessandra.depascal3@unibo.it (A.M.D.P.); ludovica.ingletto@unibo.it (L.I.); claudia.colosimo2@unibo.it (C.C.); mariasofia.montanar2@unibo.it (M.S.M.); laura.grumiro2@unibo.it (L.G.); alessand.scagliarini@unibo.it (A.S.)

**Keywords:** acute infectious gastroenteritis, enteric bacterial pathogens, enteric viral pathogens, multiplex PCR, syndromic testing, molecular diagnostics, diagnostic yield, real-world study, laboratory workflow, cost–consequence analysis

## Abstract

Acute infectious gastroenteritis (AIG) is caused by diverse bacterial and viral enteric pathogens with overlapping clinical presentations, limiting conventional pathogen-directed workflows. This retrospective before–after study evaluated the diagnostic, microbiological, operational, and economic impact of multiplex molecular syndromic testing for AIG within a regional hub-and-spoke laboratory network in Italy. Two 19-month periods were compared: a pre-implementation period based on conventional diagnostics and a post-implementation period using multiplex syndromic panels as first-line tests. Diagnostic investigations totalled 25,574 before and 24,509 after implementation. Overall test positivity increased from 5.43% to 11.59% (*p* < 0.001), with significant increases for bacterial and viral targets. Pathogen detection events increased from 1388 to 2839, including broader detection of *Campylobacter* spp., *Shigella* spp./enteroinvasive *Escherichia coli* (EIEC), *Yersinia enterocolitica*, *Aeromonas* spp., Astrovirus, Sapovirus, and Norovirus genogroups. Mean turnaround time decreased from 63 to 47 h from sample collection and from 56 to 41 h from laboratory check-in. Although direct diagnostic costs increased moderately, total laboratory costs decreased when personnel costs were included, and cost per diagnostic detection declined from €233.80 to €120.86. Multiplex syndromic testing improved microbiological detection, diagnostic efficiency, turnaround time, and laboratory organisation in routine AIG diagnosis.

## 1. Introduction

Acute infectious gastroenteritis (AIG) is one of the most common infectious syndromes worldwide and remains a major cause of morbidity across all age groups, with the greatest clinical impact in young children, older adults, and immunocompromised or frail patients [1,2]. The syndrome is characterised by the acute onset of diarrhoea and/or vomiting, often associated with abdominal pain, fever, and dehydration, and may be caused by a broad range of bacterial, viral, and parasitic pathogens [3]. Although AIG-related mortality is highest in low- and middle-income countries, the burden remains substantial in high-income settings, where AIG accounts for a large number of emergency department visits, outpatient consultations, short hospital admissions, infection-control procedures, and laboratory investigations [4,5,6,7,8,9,10]. From both a clinical and public health perspective, identifying the causative agent is important because it may support appropriate antimicrobial use, guide isolation measures, reduce unnecessary diagnostic procedures, and contribute to the recognition of seasonal or foodborne outbreaks [11,12].

The aetiological diagnosis of AIG is challenging because clinical manifestations overlap substantially among different enteric pathogens. Traditionally, laboratory diagnosis has relied on pathogen-directed workflows, including stool culture for bacterial agents, antigen detection assays for selected viruses, microscopy or specific assays for parasites, and single-target molecular tests [13]. These conventional approaches remain valuable, particularly because culture allows for antimicrobial susceptibility testing and epidemiological typing of bacterial isolates. However, they also have important limitations. Culture-based methods may require 48–72 h, have variable sensitivity depending on the organism and pre-analytical conditions, and often require selective requests based on clinical suspicion [14,15,16,17,18]. Similarly, antigen-based and single-target molecular assays investigate only a restricted number of agents and may generate fragmented diagnostic pathways when multiple pathogens need to be considered [19,20]. As a result, conventional testing may underestimate the true microbiological spectrum of AIG, especially when clinical information is incomplete or when symptoms do not clearly suggest a specific aetiology.

Multiplex molecular syndromic panels have changed the diagnostic approach to gastrointestinal infections by allowing for the simultaneous detection of multiple bacterial, viral, and parasitic targets from a single stool specimen [16,20]. These assays can improve diagnostic coverage, reduce time to aetiological identification, and detect organisms that are difficult, slow, or inconsistently investigated using conventional methods [21,22,23,24]. In paediatric and adult populations, previous studies have reported higher detection rates after implementation of gastrointestinal multiplex polymerase chain reaction (PCR) panels, together with potential effects on antimicrobial prescribing, isolation decisions, ancillary testing, and healthcare costs [11,22,23,24]. Nevertheless, the interpretation of multiplex panel results is not without controversy. Molecular detection does not necessarily indicate viable organisms or causality, particularly in the presence of colonisation, prolonged shedding, or co-detections. In addition, positive results for targets such as *Shigella* spp./EIEC or broad *Vibrio* spp. groups may require careful clinical and microbiological interpretation. Finally, concerns remain regarding the increased reagent cost of multiplex testing, the need for confirmatory culture in selected cases, and the uncertain downstream impact on patient-level outcomes [20,25].

Despite the expanding use of syndromic gastrointestinal panels, most published evidence derives from controlled evaluations, single-centre studies, or investigations focused primarily on analytical performance and patient-level clinical outcomes [24,25,26]. Less evidence is available on the real-world impact of these technologies when implemented as routine first-line tests within large regional laboratory networks. This is an important gap because, in hub-and-spoke diagnostic systems, the introduction of multiplex testing may affect not only pathogen detection, but also testing coverage, turnaround time, workflow standardisation, staffing requirements, platform utilisation, and overall laboratory efficiency. These system-level outcomes are particularly relevant for public healthcare systems, where diagnostic innovation must be evaluated not only in terms of test performance, but also in terms of organisational sustainability and microbiological value under routine operating conditions.

The present study therefore aimed to evaluate the diagnostic, microbiological, operational, and economic impact of implementing multiplex molecular syndromic testing for AIG within a regional hub-and-spoke laboratory network in Italy. Using a retrospective before–after design, we compared two consecutive 19-month periods: a pre-implementation period based on conventional diagnostic workflows and a post-implementation period in which multiplex bacterial and viral gastrointestinal (GI) panels were adopted as first-line tests. The study specifically assessed changes in diagnostic activity, overall and pathogen-specific positivity, pathogen detection spectrum, turnaround time, workflow organisation, and laboratory cost–consequence indicators. We hypothesised that syndromic testing would expand microbiological detection and improve laboratory efficiency compared with conventional, pathogen-directed workflows.

## 2. Materials and Methods

### 2.1. Study Design and Setting

This study was designed as a retrospective observational before–after study, integrated with a laboratory-based cost–consequence analysis, to evaluate the diagnostic, microbiological, operational, organisational, and economic impact of routine implementation of multiplex molecular syndromic panels for the diagnosis of acute infectious gastroenteritis (AIG).

Two observation periods of equal duration were compared. The pre-implementation period extended from January 2022 to July 2023 and was characterised by the use of conventional diagnostic workflows, including stool culture, antigen-based assays, and single-target molecular testing. The post-implementation period extended from January 2024 to July 2025 and was characterised by the adoption of multiplex molecular syndromic panels as first-line tests for bacterial and viral enteric pathogens. Each period lasted 19 months, allowing for comparison across equivalent time windows and reducing potential imbalance related to seasonal variation in gastrointestinal infections.

The study was conducted within the Greater Romagna Area laboratory network, Italy, which serves a geographic area of approximately 5000 km^2^ and an estimated population of 1.2 million inhabitants across four Local Health Authorities: Ravenna, Forlì, Cesena, and Rimini. The diagnostic system is organised according to a hub-and-spoke model, comprising a central Hub Microbiology Laboratory located in Pievesestina, Forlì-Cesena, responsible for specialised diagnostics and network-level coordination, and seven rapid-response spoke laboratories located in peripheral hospital facilities and operating 24 h a day, 7 days a week.

All microbiological analyses included in the present study were performed as part of routine diagnostic practice. No experimental diagnostic procedures were introduced for study purposes. The before–after design was therefore used to compare two real-world diagnostic and organisational models: a conventional, pathogen-directed workflow and a multiplex syndromic molecular workflow.

### 2.2. Study Population and Case Definition

The study population included all patients who underwent microbiological testing on stool specimens for suspected AIG within the Greater Romagna Area laboratory network during the two study periods. Samples originated from both community-based settings, including outpatients and emergency department visits, and hospital settings, including inpatients admitted to hub or peripheral hospitals.

For the purposes of this study, AIG was defined as an acute clinical syndrome characterised by diarrhoea and/or vomiting for which stool microbiological investigation was requested as part of routine clinical practice. No exclusion criteria were applied according to disease severity, symptom duration, care setting, age, or diagnostic outcome, in order to preserve the real-world nature of the analysis and reflect the actual diagnostic activity of the regional laboratory network.

All stool specimens were processed according to the diagnostic workflow in place during the corresponding period. During the pre-implementation phase, microbiological investigations were requested selectively according to clinical suspicion and local diagnostic practice. During the post-implementation phase, multiplex syndromic molecular testing was adopted as the first-line approach for bacterial and viral enteric pathogens. All microbiological analyses were centralised at the Hub Microbiology Laboratory in Pievesestina, with samples transported from peripheral sites according to standardised procedures.

For analytical purposes, patients were stratified by age using predefined criteria that varied according to the pathogens under investigation, in line with routine diagnostic practice and the relevant literature. For bacterial pathogens, patients were classified as ≤14 years or >14 years, corresponding to paediatric and adult populations, respectively. For viral gastrointestinal pathogens, the paediatric group was defined as ≤6 years, reflecting the higher clinical relevance and epidemiological burden of viral gastroenteritis in younger children. Cases were also stratified by healthcare setting, distinguishing community-based patients from hospitalised patients.

Because the available dataset was derived from aggregated laboratory information system outputs, the analytical unit was the diagnostic test or pathogen detection event rather than the individual patient or stool specimen. Unique specimen-level identifiers were not available; therefore, the exact number of unique stool samples and patient-level co-detections could not be reconstructed.

### 2.3. Diagnostic Workflows and Laboratory Procedures

**Figure 1:**
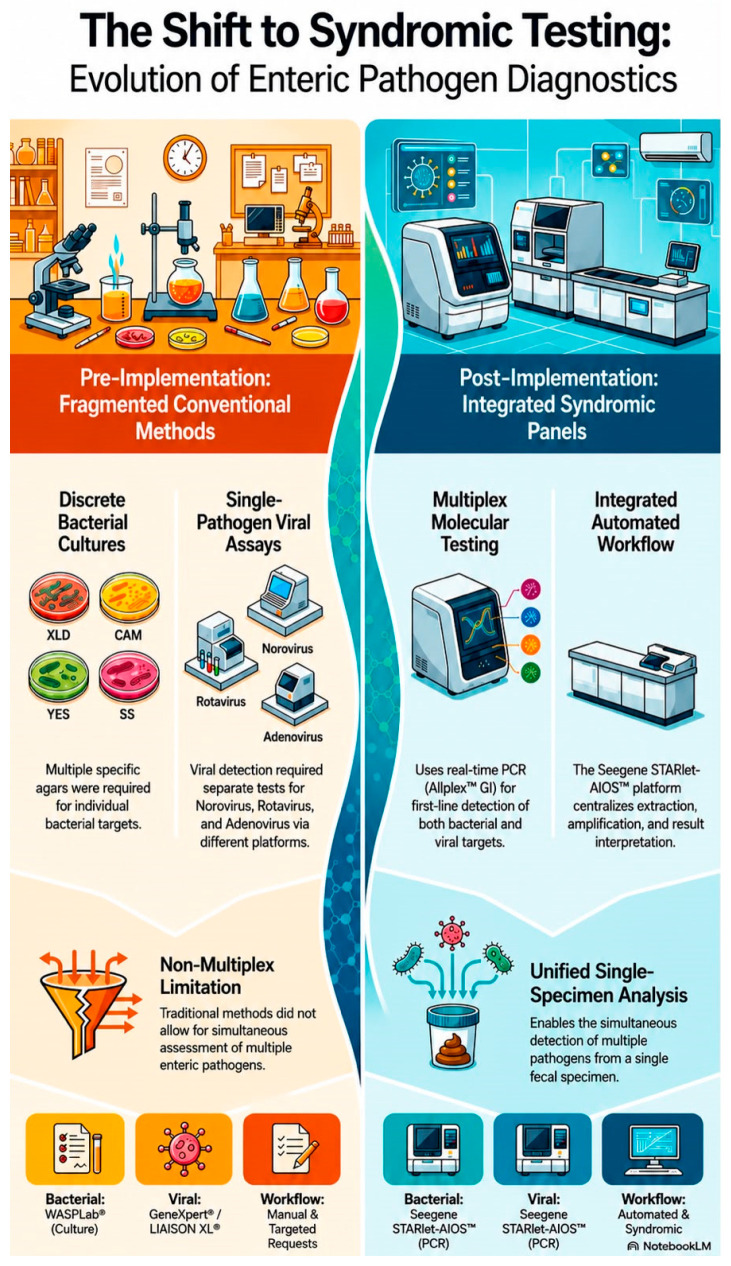
Conceptual overview of the transition from conventional to syndromic diagnostic workflows for acute infectious gastroenteritis. The figure is intended as a schematic representation of the overall diagnostic transition rather than a procedural depiction of each laboratory step. The orange sections represent the pre-implementation conventional workflow, whereas the blue sections represent the post-implementation integrated syndromic workflow. The arrows illustrate workflow progression and the convergence of multiple bacterial and viral targets into a unified single-specimen analysis. Overall, the figure illustrates the shift from a fragmented conventional approach based on separate bacterial culture-based methods and single-pathogen viral assays to an integrated syndromic molecular workflow enabling the simultaneous detection of multiple bacterial and viral enteric pathogens from a single stool specimen. The figure was created with the assistance of Google NotebookLM (Google LLC, Mountain View, CA, USA, web-based version) and subsequently reviewed and edited by the authors for scientific accuracy.

A conceptual overview of the pre-implementation and post-implementation diagnostic workflows is provided in Figure 1.

During the pre-implementation period, the diagnosis of enteric bacterial pathogens relied on conventional, pathogen-directed stool culture. Stool specimens were plated onto dedicated selective media (bioMérieux, Marcy-l’Étoile, France) according to the suspected pathogen and the clinician’s request. This request-based approach was often fragmented and variably included *Salmonella* spp., *Shigella* spp., *Campylobacter* spp., *Yersinia* spp., *Vibrio* spp., or combined requests for *Salmonella* spp. and *Shigella* spp. For the detection of *Salmonella* spp. and *Shigella* spp., specimens were first inoculated into selenite broth (bioMérieux) using the WASPLab^®^ platform (Copan Diagnostics, Brescia, Italy). After overnight incubation at 35–37 °C, the enrichment broth was subcultured onto Xylose Lysine Deoxycholate agar (XLD). When suspicious colonies were observed, further subculture was performed onto Salmonella–Shigella agar (SS) (bioMérieux), followed by incubation at 35–37 °C for 24–48 h.

*Campylobacter* spp. were cultured on Campylosel agar (bioMérieux) under microaerophilic conditions at 42 °C for up to 48 h. *Yersinia enterocolitica* was cultured on Yersinia selective agar, namely cefsulodin–irgasan–novobiocin agar (CIN agar; bioMérieux), and incubated at 32 °C for up to 48 h. Detection of *Vibrio* spp. followed a dedicated manual workflow: specimens were initially inoculated into alkaline peptone water (bioMérieux) and incubated at 35–37 °C, then subcultured onto Columbia blood agar (COS) and MacConkey agar (MCK) (bioMérieux) at 35–37 °C for up to 48 h. Bacterial isolates were identified by matrix-assisted laser desorption/ionisation time-of-flight mass spectrometry (MALDI-TOF MS) using the VITEK^®^ MS system (bioMérieux), according to the manufacturer’s instructions; VITEK^®^ cards were used when needed as an additional phenotypic method to support the differentiation of *Shigella* spp. from *Escherichia coli* [27].

During the same period, viral gastroenteritis was investigated using separate single-target assays requested according to clinical suspicion. Norovirus detection was performed using the Xpert^®^ Norovirus assay on the GeneXpert^®^ System (Cepheid, Sunnyvale, CA, USA), a fully automated qualitative nucleic acid amplification test capable of detecting and differentiating Norovirus genogroups I and II directly from stool specimens [28]. Rotavirus and Adenovirus antigen detection was performed using the LIAISON^®^ Rotavirus and LIAISON^®^ Adenovirus chemiluminescent immunoassays on the LIAISON XL^®^ automated platform (DiaSorin S.p.A., Saluggia, Italy), according to the manufacturer’s instructions [29,30,31].

In the post-implementation period, the aetiological diagnosis of AIG was performed using multiplex molecular syndromic panels as first-line tests, enabling simultaneous detection of bacterial and viral enteric pathogens from a single stool specimen. Bacterial targets were detected using the Allplex^™^ GI-Bacteria(I) Assay, and viral targets using the Allplex^™^ GI-Virus Assay (Seegene Inc., Seoul, Republic of Korea). Both assays are based on multiplex real-time PCR and were performed according to the manufacturer’s instructions using the automated Seegene STARlet-AIOS^™^ platform, which integrates the main steps of the analytical workflow [32]. Nucleic acid extraction was performed using the STARMag^™^ 96 × 4 Universal Cartridge Kit (Seegene Inc., Seoul, Republic of Korea). Amplification and real-time detection were carried out on the CFX96 Touch Deep Well Real-Time PCR Detection System (Bio-Rad Laboratories, Hercules, CA, USA), and results were interpreted using Seegene Viewer software version 3.30.000 [33,34].

Throughout both study periods, microbiological analyses were conducted according to the routine diagnostic protocols in place, with no changes in clinical test-request criteria, decision thresholds, or reporting practices.

### 2.4. Outcome Measures and Cost–Consequence Analysis

Study outcomes were defined a priori to evaluate the diagnostic, microbiological, operational, organisational, and economic impact of implementing multiplex molecular syndromic panels compared with the previous conventional diagnostic workflow. These outcomes were grouped into four domains and analysed separately for the pre-implementation and post-implementation periods.

Diagnostic and microbiological outcomes included overall test positivity, category-specific test positivity, pathogen-specific detection rates, and absolute pathogen detection counts. Given the aggregated structure of the available dataset, analyses were conducted at the level of the diagnostic test or pathogen detection event rather than at the level of the individual patient or stool specimen. Overall test positivity was defined as the proportion of diagnostic tests yielding at least one positive result among all tests performed during the corresponding period. Category-specific positivity was calculated separately for bacterial and viral investigations, with additional stratification by healthcare setting and age group. For each individual target, the pathogen-specific detection rate corresponded to the proportion of positive results among all tests in which that target was actively investigated. To improve comparability between the two diagnostic models, pathogen-specific analyses were restricted to bacterial and viral targets shared across both periods. Absolute pathogen detection counts were also recorded to describe changes in the spectrum of enteric pathogens identified after implementation of multiplex syndromic testing. Because unique specimen-level identifiers were not available, co-detections within the same stool specimen could not be reconstructed, and sample-level or patient-level diagnostic yield could not be estimated.

Operational outcomes included turnaround time (TAT) and diagnostic throughput. TAT was expressed in hours and evaluated using two predefined intervals: from sample collection to report validation, and from laboratory check-in to report validation. Values were derived from aggregated temporal information available in the laboratory information system and summarised as means for each study period. As specimen-level timestamps were unavailable, intra-period variability could not be assessed, and no inferential statistical testing was performed for TAT comparisons. Diagnostic throughput was defined as the number of diagnostic tests processed per unit of time and expressed as the mean number of tests processed per month, using the formula Throughput = N_tests_/T, where N_tests_ represents the total number of diagnostic tests performed during the corresponding period and T the duration of the observation window in months.

Organisational outcomes included laboratory space utilisation, workflow restructuring, instrumentation requirements, manual handling, degree of workflow standardisation, and modelled staffing configuration. These indicators were assessed descriptively by comparing the conventional and syndromic workflows across the two periods. The evaluation considered the number and type of analytical steps, the use of culture-based versus molecular platforms, the requirement for downstream identification, the number of platforms involved, the infrastructure needed to support each workflow, and the overall complexity of the diagnostic pathway. Organisational outcomes were analysed qualitatively and comparatively, without inferential statistical testing.

Economic outcomes were assessed through a laboratory-based cost–consequence analysis comparing the conventional and syndromic diagnostic strategies. The analysis was conducted from the laboratory perspective and included both direct diagnostic costs and personnel costs. Direct diagnostic costs comprised instrument-related expenditure, reagents, consumables, culture media, enrichment broths, assay-specific materials, and, when applicable, isolate identification. Personnel costs were calculated separately and then combined with direct diagnostic costs to obtain overall laboratory costs. The costing framework and formulas used for the analysis are summarised in Supplementary Table S1.

For the pre-implementation period, conventional bacterial diagnostics were costed using a bottom-up approach based on the pathways actually used in routine practice and defined by clinical request and analytical process. These included routine stool culture with negative results, routine stool culture with positive or suspicious results requiring MALDI-TOF MS identification, selective testing for *Yersinia* spp., and selective testing for *Vibrio* spp. To enhance generalisability, cost estimates were based on national values rather than laboratory-specific figures. Instrument-related expenditure, including leasing and service fees, was allocated over the 19-month observation window according to actual use, weighted by attributing 33% of total platform utilisation to gastroenterological diagnostics, and distributed over the total number of samples processed on the instrument. This allocation was applied on a per-sample basis and was not multiplied by the number of inoculation steps, as it already reflected total platform utilisation. Culture media and enrichment reagent costs were assigned to each pathway according to the materials actually used, including selective and differential media and enrichment broths. In routine stool culture, all samples undergoing the standard culture workflow received a common base cost, whereas MALDI-TOF MS identification was added only as an incremental cost for positive or suspicious cultures requiring isolate identification. WASPLab^®^ costs were not included for the *Vibrio* spp. pathway because sample processing was performed manually.

Conventional viral diagnostics were modelled using the same instrument-cost allocation approach and comprised three single-target pathways: Norovirus, Rotavirus, and Adenovirus. Norovirus testing was modelled on a four-module GeneXpert^®^ System, reflecting a realistic routine configuration for single-target molecular testing in clinical microbiology. This configuration was selected because the GeneXpert IV provides a compact, scalable, random-access platform suitable for limited parallel testing volumes. Larger high-capacity configurations were not considered, as they are intended for centralised or high-throughput settings and would likely underestimate the instrument-related cost per test in the conventional scenario. For each viral pathway, unit cost was calculated as the sum of instrument-related expenditure and assay-specific reagents/consumables.

During the post-implementation period, syndromic molecular diagnostics were costed using the same bottom-up logic, but through a unified framework applied to both bacterial and viral panels. Unlike the pre-implementation model, in which costs were distributed across multiple heterogeneous workflows, multiplex syndromic testing standardised the analytical process and allowed costs to be estimated through a common structure irrespective of pathogen category. Unit cost per test included instrument-related expenditure and panel-specific reagents/consumables, covering sample processing, nucleic acid extraction, amplification, and detection. Leasing and service fees were allocated over the observation window according to actual use, again attributing 33% of total platform utilisation to gastroenterological diagnostics. Reagent and consumable costs were assigned to each panel on the basis of national values. No routine downstream procedures, such as isolate identification or confirmatory phenotypic work-up, were included in the syndromic workflow.

Personnel costs were estimated from the perspective of the Italian National Health Service using a top-down approach based on the standard annual cost of the main professional roles involved in the diagnostic process, namely Biomedical Laboratory Technicians, D3 category, and Clinical Biologists [35,36]. Annual costs were derived from national collective labour agreements, including gross salary and employer-related contributions, normalised to the 19-month observation window, and informed by Agenzia per la Rappresentanza Negoziale delle Pubbliche Amministrazioni (ARAN) public-sector remuneration data [37]. Since these professional roles were not exclusively dedicated to gastroenterological diagnostics, the allocated amount was adjusted using a multiplicative factor of 0.75, representing the proportion of working time effectively attributable to the activities under investigation. This assumption was consistent with standard principles of health economic evaluation and time-driven activity-based costing, whereby personnel costs are allocated proportionally to the time or activity share attributable to the process of interest, in order to avoid overestimation of pathway-specific costs [38,39,40].

Three derived economic indicators were used to compare the two strategies. First, cost per diagnostic detection was calculated as an indicator of the economic efficiency of pathogen identification, using the total number of pathogen detection events observed in each period. Second, cost composition analysis described the proportional contribution of the main cost categories, namely instrument-related costs, reagent/consumable costs, and personnel costs, to total laboratory expenditure. Finally, economic efficiency relative to TAT reduction was expressed as the cost or saving per hour of TAT saved, using mean TAT estimates for the two periods. This indicator was assessed from two complementary perspectives: overall laboratory costs, including instrument-related costs, reagents/consumables, and personnel costs, and direct diagnostic costs only, including instrument-related costs and reagents/consumables but excluding personnel. Negative values indicated net savings associated with TAT reduction, whereas positive values indicated additional expenditure per hour saved.

### 2.5. Statistical Analysis, Data Management, and Ethics

Statistical analyses were performed to compare outcomes between the pre-implementation period, from January 2022 to July 2023, and the post-implementation period, from January 2024 to July 2025. Categorical variables were summarised as counts and percentages. Test positivity and pathogen-specific detection rates were compared between periods using the Pearson χ^2^ test, while Fisher’s exact test was applied when expected cell counts were <5. Analyses were stratified a priori by healthcare setting, distinguishing community-based from hospitalised patients, and by age group according to predefined clinical and epidemiological cut-offs: ≤14 versus >14 years for bacterial pathogens and ≤6 versus >6 years for viral pathogens.

All tests were two-sided. Statistical significance was interpreted using the following thresholds: *p* < 0.001, *p* < 0.01, *p* < 0.05, and ns, not significant. Operational indicators available only at the aggregated level, including mean TAT from sample collection and from laboratory check-in, were analysed descriptively by study period. Because of the aggregated structure of the dataset, no patient-level inferential testing, time-to-event modelling, or multivariable adjustment was performed. Accordingly, statistical comparisons should be interpreted as associations between study period and observed outcomes, reflecting both changes in diagnostic strategy and differences in testing coverage. All analyses were performed using Stata version 19 (StataCorp LLC, College Station, TX, USA).

Study data were derived from the laboratory information system (LIS) and electronic medical records (EMRs). The LIS was used to extract microbiological test activity, sample acceptance and reporting dates, and diagnostic results, whereas the EMRs were used only to retrieve patient age and healthcare setting, classified as community-based or hospitalised. No direct patient identifiers, sensitive clinical information, diagnoses, comorbidities, treatments, or individual patient outcomes were collected or analysed. All data were processed in aggregated and anonymised form, in accordance with applicable personal data protection regulations, including the European Union General Data Protection Regulation (EU GDPR 2016/679).

The study was submitted to and approved by the local ethics committee, the Comitato Etico della Romagna (CET-CEROM; Reg. sperimentazioni n. 3912; Prot. 3372/2025), with approval issued following the asynchronous session convened on 1 August 2025. Given the retrospective observational design and the use of anonymised data generated as part of routine clinical practice, individual informed consent was not required. The study was conducted in accordance with the ethical principles of the Declaration of Helsinki.

## 3. Results

### 3.1. Diagnostic Coverage and Testing Activity

Implementation of multiplex syndromic molecular testing substantially expanded the aetiological spectrum investigated for acute infectious gastroenteritis. In the pre-implementation period, pathogen detection was based on targeted investigations driven by clinical suspicion, with bacterial testing focused mainly on conventional stool culture and viral testing limited to selected single-target assays. After implementation, the diagnostic approach shifted to systematic assessment of a broader panel of bacterial and viral enteric pathogens from each stool specimen.

**Figure 2:**
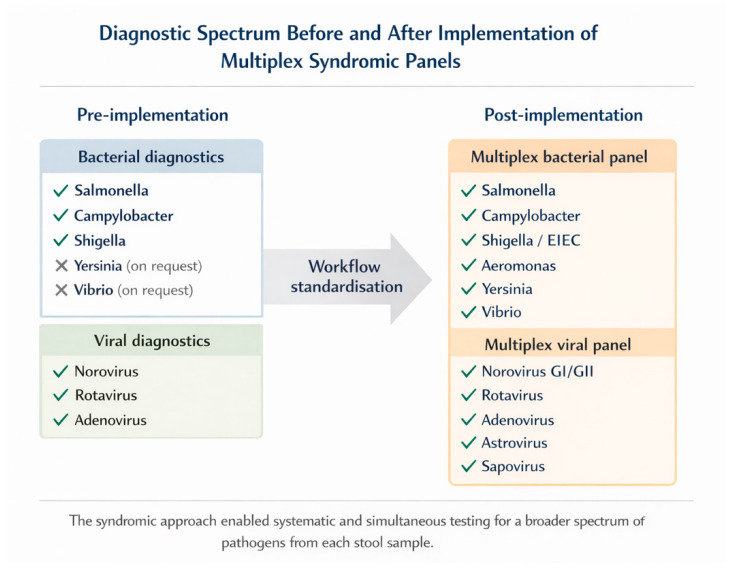
Diagnostic spectrum before and after implementation of multiplex syndromic panels. The figure illustrates the transition from a targeted, partly request-based conventional workflow to a standardised syndromic approach enabling systematic and simultaneous testing for a broader spectrum of bacterial and viral enteric pathogens from each stool specimen. Green check marks (✓) indicate pathogens routinely included in the diagnostic workflow, whereas grey crosses (×) indicate pathogens investigated only upon specific clinical request.

As shown in Figure 2, the post-implementation workflow routinely included bacterial targets such as *Aeromonas* spp., *Campylobacter* spp., *Salmonella* spp., *Shigella* spp./EIEC, *Yersinia enterocolitica*, and *Vibrio* spp., together with viral targets including Norovirus GI/GII, Rotavirus, Adenovirus, Astrovirus, and Sapovirus. This transition replaced a selective and partly fragmented diagnostic model with a standardised multiplex strategy, increasing pathogen coverage across both bacterial and viral categories. A detailed comparison of diagnostic targets and methods before and after implementation is reported in Supplementary Table S2.

During the pre-implementation period, 25,574 diagnostic investigations were performed, including 15,987 bacterial tests (62.5%) and 9587 viral tests (37.5%). In the post-implementation period, overall diagnostic activity slightly decreased to 24,509 tests (−4.2%), but the distribution by pathogen category changed substantially. Bacterial testing increased to 19,809 investigations (+23.9%), whereas viral testing decreased to 4700 investigations (−51.0%) (Table 1).

Testing volumes also varied by healthcare setting. Before implementation, bacterial investigations were performed predominantly in community-based patients (10,987; 68.7%) compared with hospitalised patients (5000; 31.3%). After implementation, bacterial diagnostic activity increased in both settings, reaching 13,447 tests in the community (+22.4%) and 6362 among hospitalised patients (+27.2%), while maintaining a similar proportional distribution. Conversely, viral investigations decreased overall, with a greater reduction among hospitalised patients (−60.4%) than in the community setting (−41.5%) (Supplementary Table S3).

At target level, viral testing showed an apparently divergent pattern. Although the total number of viral tests decreased after implementation, the number of investigations in which specific viral targets were assessed increased for Norovirus (+83.2%), Adenovirus (+38.0%), and Rotavirus (+30.0%). This reflected the transition from selective single-target assays to a multiplex panel-based strategy, in which multiple viral agents were evaluated simultaneously within each test (Supplementary Table S4).

Overall, multiplex syndromic testing reorganised diagnostic activity rather than simply increasing or decreasing test volumes. The post-implementation period was characterised by expanded bacterial testing and broader viral target assessment despite fewer viral test orders.

### 3.2. Test Positivity and Pathogen Detection

The expansion of diagnostic coverage was accompanied by a marked increase in pathogen detection events. In the pre-implementation period, 714 bacterial and 674 viral positive detections were documented. Bacterial detections were mainly attributable to *Campylobacter* spp. (*n* = 470) and *Salmonella* spp. (*n* = 239), with only sporadic detection of *Shigella* spp. (*n* = 1) and *Yersinia enterocolitica* (*n* = 4), and no confirmed detection of *Vibrio* spp. Viral detections included Norovirus (*n* = 382), Adenovirus (*n* = 204), and Rotavirus (*n* = 88) (Supplementary Table S5). Age-stratified absolute counts are reported in Supplementary Table S6.

After implementation, positive detections increased to 1765 for bacterial pathogens and 1074 for viral pathogens. The most frequently detected bacterial agents were *Campylobacter* spp. (*n* = 835) and *Salmonella* spp. (*n* = 297), followed by *Aeromonas* spp. (*n* = 499), *Yersinia enterocolitica* (*n* = 68), *Shigella* spp./EIEC (*n* = 60), and *Vibrio* spp. (*n* = 6). Among viral pathogens, Norovirus GI/GII was the most frequently detected target after implementation, with 470 detection events. Rotavirus accounted for 221 detections, while newly included viral targets contributed substantially to the post-implementation diagnostic spectrum, with 166 Sapovirus and 96 Astrovirus detections. Adenovirus detections decreased to 121 compared with the pre-implementation period (Supplementary Table S7). Age-stratified post-implementation detections are reported in Supplementary Table S8A,B, while the distribution by healthcare setting is shown in Supplementary Table S8C. Overall, 2839 positive detections were documented after implementation, with more detection events recorded in community-based patients than among hospitalised patients.

When the analysis was restricted to targets investigated in both study periods, shared bacterial detections increased from 714 to 1266, and shared viral detections from 674 to 812 (Supplementary Table S9). Detection counts increased for *Campylobacter* spp., *Salmonella* spp., *Shigella* spp./EIEC, *Yersinia enterocolitica*, Norovirus, and Rotavirus, whereas Adenovirus detections decreased. These findings indicate that multiplex syndromic testing increased overall pathogen detection while also broadening the detectable aetiological spectrum.

Consistently, overall test positivity increased from 5.43% in the pre-implementation period to 11.59% after implementation (χ^2^ test, *p* < 0.001; Table 2). Category-specific positivity increased significantly for both bacterial pathogens, from 4.47% to 8.91%, and viral pathogens, from 7.03% to 22.85% (χ^2^ test, *p* < 0.001 for both comparisons; Table 2; Supplementary Figure S1). The increase in positivity despite a slight reduction in overall diagnostic activity supports an improvement in diagnostic yield at test level.

### 3.3. Stratified Positivity and Pathogen-Specific Detection Rates

The increase in test positivity was maintained across healthcare settings and age groups. By healthcare setting, overall positivity increased from 5.08% to 11.11% in community-based patients and from 5.99% to 12.51% among hospitalised patients (χ^2^ test, *p* < 0.001 for both comparisons). Category-specific analyses showed the same direction of change: bacterial positivity increased from 4.00% to 8.50% in the community setting and from 5.50% to 9.80% among hospitalised patients, while viral positivity increased from 7.54% to 23.73% and from 6.52% to 21.56%, respectively (χ^2^ test, *p* < 0.001 for all comparisons; Supplementary Table S10; Supplementary Figure S2).

Age-stratified analyses confirmed this pattern. For bacterial pathogens, positivity increased from 7.21% to 12.02% among patients aged ≤14 years and from 3.64% to 8.12% among those aged >14 years (χ^2^ test, *p* < 0.001 for both comparisons), with consistent increases across both community and hospital settings (Supplementary Table S11; Supplementary Figure S3). For viral pathogens, using the predefined age stratification of ≤6 versus >6 years, positivity increased from 9.53% to 34.95% in younger children and from 4.20% to 13.08% in older individuals (χ^2^ test, *p* < 0.001 for both comparisons). The highest post-implementation viral positivity was observed among children aged ≤6 years, both in the community setting (38.08%) and among hospitalised patients (30.72%) (Supplementary Table S12).

When restricted to shared targets investigated in both periods, pathogen-specific detection rates showed heterogeneous changes (Table 3). Among bacterial targets, detection rates increased for *Campylobacter* spp., from 3.16% to 4.22%, and for *Shigella* spp./EIEC, from 0.01% to 0.30% (χ^2^ test or Fisher’s exact test, *p* < 0.001). *Vibrio* spp. were not detected before implementation, whereas six detections were recorded after implementation. The multiplex panel enabled detection of a broader range of *Vibrio* spp., whereas the pre-implementation culture-based approach targeted primarily *Vibrio cholerae*; notably, no *V. cholerae* cases were identified in either period, as confirmed by culture. By contrast, *Yersinia enterocolitica* remained numerically stable, while *Salmonella* spp. showed a small numerical decrease, from 1.61% to 1.50%, despite statistical significance likely related to the large sample size (Supplementary Figure S4).

Among shared viral targets, divergent trends were observed. Norovirus detection rate decreased from 14.89% to 10.00%, and Adenovirus from 5.99% to 2.57%, whereas Rotavirus increased from 2.43% to 4.70% (χ^2^ test, *p* < 0.001 for all comparisons; Supplementary Figure S5). Additional pathogen-specific stratification by healthcare setting and age group is reported in Supplementary Tables S13 and S14 and Supplementary Figures S6–S9. These analyses confirmed that pathogen-specific changes were not uniform across strata. In community-based patients, increases were mainly observed for *Campylobacter* spp. and *Shigella* spp./EIEC, whereas *Salmonella* spp. and *Yersinia enterocolitica* remained broadly stable. Among hospitalised patients, *Shigella* spp./EIEC increased, while no significant changes were observed for *Salmonella* spp. or *Campylobacter* spp. Shared viral targets also showed setting-specific differences, with Norovirus and Adenovirus decreasing mainly in the community setting and Rotavirus increasing particularly among hospitalised patients. Age-stratified analyses showed increases in *Campylobacter* spp. and *Shigella* spp./EIEC in both paediatric and adult groups, while viral trends were more age-dependent, with Rotavirus increasing mainly in children aged ≤6 years and Adenovirus decreasing predominantly among individuals aged >6 years.

Overall, these findings indicate that the impact of syndromic testing varied by pathogen, setting, and age group, with a consistent increase in aggregate positivity but heterogeneous pathogen-specific shifts reflecting both expanded coverage and changes in testing denominators.

### 3.4. Turnaround Time and Workflow Reorganisation

Implementation of multiplex syndromic panels was associated with a marked reduction in diagnostic turnaround time and with substantial reorganisation of laboratory workflow. Mean TAT from sample collection decreased from 63 h in the pre-implementation period to 47 h after implementation, corresponding to a 25.4% reduction. Similarly, mean TAT from laboratory check-in decreased from 56 h to 41 h, corresponding to a 26.8% reduction (Table 4; Supplementary Figure S10).

Given the identical duration of the two observation periods, overall diagnostic throughput showed a slight decrease, from 1346 to 1290 tests per month (−4.2%). This reflected divergent changes by pathogen category: bacterial throughput increased from 841 to 1043 tests per month (+23.9%), whereas viral throughput decreased from 505 to 247 tests per month (−51.1%). Thus, the reduction in TAT occurred despite stable overall diagnostic workload, indicating that the observed improvement was mainly related to workflow reorganisation rather than increased testing volume.

Organisational differences between the two diagnostic models are summarised in Supplementary Table S15. The pre-implementation workflow was characterised by separate bacterial and viral pathways, culture-based processing, selective enrichment and incubation steps, manual plate reading, downstream isolate identification, and the coordinated use of multiple analytical platforms. Viral diagnostics were similarly fragmented across target-specific assays and dedicated instruments. This configuration required extensive manual handling, multiple laboratory areas, and a modelled staffing configuration of three Biomedical Laboratory Technicians and one Clinical Biologist.

After implementation, bacterial and viral diagnostics were incorporated into a standardised molecular workflow, reducing the number of operational steps, limiting manual handling, and eliminating routine downstream phenotypic identification. The post-implementation configuration required one Biomedical Laboratory Technician and one Clinical Biologist and reduced infrastructure needs by consolidating diagnostics on an integrated molecular platform. Overall, syndromic testing shortened reporting times while simplifying laboratory organisation, staffing requirements, and platform management.

### 3.5. Cost–Consequence Analysis

The implementation of multiplex syndromic testing was associated with a moderate increase in direct diagnostic expenditure but lower overall laboratory costs when personnel costs were included. In the pre-implementation period, the estimated direct diagnostic cost of conventional testing was €324,538.90, of which €111,637.50 (34.4%) was attributable to bacterial diagnostics and €212,901.40 (65.6%) to viral diagnostics. After implementation, direct diagnostic costs increased to €343,126.00, with bacterial syndromic panels accounting for €277,326.00 (80.8%) and viral panels for €65,800.00 (19.2%). This corresponded to an absolute increase of €18,587.10 (+5.7%). The conventional workflow was therefore economically driven predominantly by single-target viral testing, whereas the post-implementation cost structure was concentrated mainly in bacterial syndromic testing, reflecting both the higher number of bacterial panels performed and the standardised structure of the multiplex workflow (Supplementary Tables S16 and S17).

Estimated personnel costs decreased from €249,375.00 to €154,375.00, corresponding to an absolute reduction of €95,000.00 (−38.1%). This reduction reflected the different staffing configurations modelled across the two periods, with the conventional workflow requiring three Biomedical Laboratory Technicians and one Clinical Biologist, compared with one Biomedical Laboratory Technician and one Clinical Biologist in the syndromic workflow (Supplementary Table S18). The annual cost assumptions used for these calculations were based on national collective labour agreements and ARAN remuneration data, as specified in the Methods Section [35,36,37,38,39,40].

Economic efficiency improved substantially when costs were related to pathogen detection. Total pathogen detection events increased from 1388 to 2839, while the direct diagnostic cost per detection decreased from €233.80 to €120.86, corresponding to an absolute reduction of €112.94 per detection (−48.3%) (Supplementary Table S19). Thus, the higher direct diagnostic expenditure associated with syndromic testing was offset by the larger number of pathogens detected.

Cost composition analysis showed a redistribution of the main cost components. Instrument-related costs decreased from €113,369.40 to €49,018.00 (−56.8%), and personnel costs from €249,375.00 to €154,375.00 (−38.1%). By contrast, reagent and consumable costs increased from €211,169.50 to €294,108.00 (+39.3%). Overall, total laboratory costs decreased from €573,913.90 to €497,501.00, corresponding to an absolute reduction of €76,412.90 (−13.3%) (Supplementary Table S20).

When turnaround time reduction was analysed in relation to costs, the interpretation differed according to the costing perspective. Considering overall laboratory costs, syndromic testing was associated with net savings of €4775.81 per hour saved from sample collection and €5094.19 per hour saved from laboratory check-in (Supplementary Table S21). Conversely, when only direct diagnostic costs were considered, the corresponding values indicated additional expenditure of €1161.69 and €1239.14 per hour saved, respectively (Supplementary Table S22).

Taken together, these findings indicate that syndromic testing shortened TAT and increased direct assay-related expenditure, but improved overall economic efficiency through higher pathogen detection, reduced personnel requirements, and lower total laboratory costs. A summary of the main economic and operational indicators is provided in Table 5.

## 4. Discussion

Acute infectious gastroenteritis (AIG) remains one of the most common infectious syndromes and one of the most operationally burdensome conditions in routine clinical practice, particularly in paediatric populations, frail patients, and high-turnover care settings [22]. In this context, the value of microbiological diagnosis lies not only in pathogen identification, but also in its ability to reduce early aetiological uncertainty in a clinically heterogeneous condition, with potential implications for patient isolation, antimicrobial prescribing, the need for further investigations, and the overall use of healthcare resources [41].

The present real-world study, conducted within a regional hub-and-spoke laboratory network, shows that routine implementation of multiplex molecular syndromic panels for AIG diagnosis was associated with broader diagnostic coverage, higher test positivity, shorter turnaround time (TAT), and marked simplification of laboratory workflow compared with the previous conventional approach. Overall positivity increased from 5.43% to 11.59%, with significant increases in both bacterial and viral investigations. In parallel, mean TAT decreased from 63 to 47 h from sample collection and from 56 to 41 h from laboratory check-in. Taken together, these findings indicate that the value of syndromic diagnostics should not be interpreted exclusively in terms of analytical performance or unit test cost, but also in relation to its broader effects on laboratory organisation, diagnostic efficiency, and the management of patients with suspected infectious gastroenteritis.

The increase in microbiological detection should be interpreted as the combined result of molecular detection and, more importantly, a change in the diagnostic paradigm. Before implementation, testing depended on targeted requests guided by clinical suspicion and sequential investigations; after implementation, a broader range of bacterial and viral agents was assessed systematically from each stool specimen. This transition from fragmented, target-oriented requesting to a standardised syndromic model is particularly relevant in AIG, where clinical manifestations overlap substantially across bacterial and viral aetiologies and symptom-based prediction of the causative agent is often unreliable. By reducing dependence on selective test requests, multiplex panels may limit underdiagnosis and improve consistency across the diagnostic pathway.

Pathogen-specific findings require a more cautious interpretation. The increased detection observed for targets such as *Campylobacter* spp., *Shigella* spp./EIEC, *Yersinia enterocolitica*, and Rotavirus is consistent with the expected advantages of molecular methods over conventional approaches, particularly for pathogens that may be less consistently captured by culture or antigen-based assays. Conversely, the relative stability of *Salmonella* spp. detection suggests that the benefit of syndromic panels is not uniform across all targets, but depends on the performance of the previous comparator method, the biology of the organism, and the diagnostic denominator used for comparison. This differentiated pattern strengthens the interpretation of the study, as syndromic testing did not generate an indiscriminate increase in positivity across all pathogens.

The reduction in detection rates observed for some shared viral targets, particularly Norovirus and Adenovirus, should also be interpreted in light of the changed testing model. In the pre-implementation period, these targets were investigated selectively in populations with a higher pre-test probability; after implementation, they were assessed systematically in a broader and less selected population. Under these conditions, a decrease in target-specific positivity rate may coexist with stable or increased absolute detections, as observed for Norovirus. For Adenovirus, the reduction involved both relative and absolute detections, suggesting a more complex epidemiological or testing-related pattern. Overall, pathogen-specific comparisons should therefore be viewed as reflecting a change in diagnostic strategy rather than a direct head-to-head comparison of analytical accuracy between individual methods.

Beyond pathogen detection, one of the most relevant contributions of this study concerns the operational and organisational impact of syndromic testing. The conventional workflow required multiple analytical streams, culture-based processing, selective incubations, manual plate reading, subculture, phenotypic identification, and separate viral platforms. Each additional step represented a potential source of delay, variability, and operational vulnerability. By contrast, the syndromic workflow incorporated bacterial and viral testing into a more standardised molecular process, reducing manual handling, limiting the need for multiple platforms, and eliminating routine downstream phenotypic identification. In a regional laboratory network where samples from different care settings converge on a central hub, this simplification may be particularly valuable because it reduces operational heterogeneity and improves reproducibility.

The observed shortening of TAT should be interpreted within this broader organisational framework. From a laboratory perspective, reduced TAT reflects improved process efficiency; from a clinical and system-level perspective, earlier aetiological clarification may support decisions on isolation, further diagnostic work-up, empirical antibiotic use, emergency department flow, admission appropriateness, and duration of observation. Although these downstream outcomes were not directly measured, a reduction of approximately 16 h from sample collection and 15 h from laboratory check-in is likely to be meaningful in high-volume settings and in vulnerable populations, including young children, older adults, and frail patients.

The COVID-19 pandemic provides a useful conceptual parallel. It clearly showed that the value of rapid and reliable diagnostics does not depend only on analytical accuracy or the cost of the individual test, but also on the ability to support timely clinical and organisational decisions, including patient cohorting, bed allocation, isolation measures, and infection control [42]. Although AIG and COVID-19 differ substantially in epidemiology and clinical severity, the underlying organisational principle is transferable: when an infectious syndrome is common, clinically nonspecific, and managed at scale, diagnostic turnaround time becomes a system-level variable rather than a purely laboratory metric.

This perspective is also relevant economically. Limiting the comparison to assay price or immediate test cost may underestimate the value of syndromic testing. In this study, direct diagnostic costs increased only moderately, from €324,538.90 to €343,126.00. However, this increase was accompanied by a substantial rise in pathogen detection events and a reduction in cost per detection from €233.80 to €120.86. When personnel costs were included, total laboratory costs decreased from €573,913.90 to €497,501.00, with a 38.1% reduction in personnel expenditure. These findings suggest that workflow standardisation and reduced organisational complexity may generate economic benefits that are not captured by reagent cost alone.

From the perspective of the National Health Service, the economic value of earlier and more standardised diagnosis should therefore be placed within a broader cost framework. Potential downstream effects may include avoidable hospital admissions, reduced length of stay, shorter emergency department observation, fewer additional diagnostic investigations, reduced inappropriate empirical treatment, optimisation of isolation measures, and improved bed management. Because the present analysis was restricted to the laboratory perspective, these broader clinical and organisational consequences could not be quantified. The economic findings should therefore be regarded as conservative, as they do not capture the full spectrum of possible downstream savings.

Several limitations should be acknowledged, while also outlining directions for further research. First, the retrospective before–after design does not allow for definitive causal attribution of all observed changes to syndromic testing alone, as temporal trends, variation in pathogen circulation, clinical requesting behaviour, or organisational factors may have contributed to differences between periods. Second, the use of aggregated laboratory data prevented patient-level analyses, reconstruction of unique stool samples, assessment of co-detections, and evaluation of individual clinical outcomes. Third, pathogen-specific comparisons should be interpreted with caution, as they reflect the transition from selective, clinically guided testing to systematic multiplex assessment rather than direct head-to-head estimates of analytical performance. Finally, the economic analysis was limited to the laboratory perspective and did not include downstream healthcare costs, such as length of stay, isolation duration, antimicrobial prescribing changes, additional investigations, or patient-flow outcomes. These limitations identify a clear next step: future patient-level studies should integrate microbiological results with clinical, organisational, and economic endpoints to quantify more directly the impact of syndromic testing on patient management and healthcare-system efficiency.

Despite these limitations, the study provides relevant real-world evidence that implementation of syndromic diagnostics for AIG within a regional public laboratory network may generate benefits extending beyond the analytical dimension. The value of this strategy lies not only in broader diagnostic coverage and shorter TAT, but also in its ability to reorganise the diagnostic system, support earlier and more standardised decision-making, and potentially reduce downstream healthcare costs not visible from the laboratory budget alone. Overall, our findings suggest that, in modern AIG management, syndromic diagnostics should be regarded not merely as a technological innovation, but as a strategic tool for improving clinical, organisational, and economic efficiency within public healthcare systems.

## 5. Conclusions

Within a regional hub-and-spoke laboratory network, routine implementation of multiplex molecular syndromic panels for acute infectious gastroenteritis was associated with broader aetiological coverage, higher test positivity (5.43% vs. 11.59%), and shorter turnaround times, both from sample collection (63 vs. 47 h) and from laboratory check-in (56 vs. 41 h). The syndromic strategy also simplified the diagnostic workflow by reducing analytical complexity, manual handling, and platform fragmentation. Although direct diagnostic costs increased moderately, overall efficiency improved, as shown by the lower cost per diagnostic detection (€233.80 vs. €120.86) and the reduction in total laboratory costs once personnel costs were included. These findings indicate that the value of syndromic diagnostics extends beyond broader or faster testing alone, supporting a more timely, standardised, and organisationally efficient approach to routine AIG diagnosis. Overall, syndromic diagnostics for AIG should be regarded not only as a technological innovation, but as a strategic tool for optimising diagnostic pathways within public healthcare systems. Further patient-level studies are needed to define its direct impact on clinical outcomes, healthcare resource use, and downstream costs.

## Figures and Tables

**Table 1:** Overall diagnostic activity for acute infectious gastroenteritis during the pre- and post-implementation periods.

Study Period	Total Tests (*n*)	Bacterial Tests (*n*)	Viral Tests (*n*)
Pre-implementation (Jan 2022–Jul 2023)	25,574	15,987	9587
Post-implementation (Jan 2024–Jul 2025)	24,509	19,809	4700
Relative change (%)	−4.2%	+23.9%	−51.0%

Note: Data represent aggregated counts of diagnostic tests performed. The number of unique stool samples could not be reconstructed from the available dataset.

**Table 2:** Overall and category-specific test positivity before and after implementation of multiplex syndromic panels.

Analysis Level	Study Period	Tests Performed, *n*	Positive Detections, *n*	Positivity Rate (%)	Statistical Test	*p* Value	Significance
Overall	PRE	25,574	1388	5.43	χ^2^ test	<0.001	***
	POST	24,509	2839	11.59			
Bacterial pathogens	PRE	15,987	714	4.47	χ^2^ test	<0.001	***
	POST	19,809	1765	8.91			
Viral pathogens	PRE	9587	674	7.03	χ^2^ test	<0.001	***
	POST	4700	1074	22.85			

Note: Test positivity was defined as the proportion of diagnostic tests yielding at least one pathogen detection within each study period or pathogen category. Comparisons between pre- and post-implementation periods were performed using the χ^2^ test for independence. Counts refer to aggregated test-level detection events and do not correspond to unique stool samples or individual patients. Statistical significance levels are reported as *** *p* < 0.001.

**Table 3:** Pathogen-specific detection rates for shared enteric targets before and after implementation of multiplex syndromic panels.

Pathogen Category	Pathogen	Study Period	Target-Specific Tests, *n*	Positive Detections, *n*	Positivity Rate (%)	Statistical Test	*p* Value	Significance
Bacterial	*Salmonella* spp.	PRE	14,865	239	1.61	χ^2^ test	<0.001	***
		POST	19,809	297	1.50			
	*Campylobacter* spp.	PRE	14,865	470	3.16	χ^2^ test	<0.001	***
		POST	19,809	835	4.22			
	*Shigella* spp.	PRE	14,865	1	0.01	Fisher’s exact	<0.001	***
		POST	19,809	60	0.30			
	*Yersinia enterocolitica*	PRE	1068	4	0.37	Fisher’s exact	<0.001	***
		POST	19,809	68	0.34			
	*Vibrio* spp.	PRE	54	0	0.00	Fisher’s exact	<0.001	***
		POST	19,809	6	0.03			
Viral	Norovirus	PRE	2566	382	14.89	χ^2^ test	<0.001	***
		POST	4700	470	10.00			
	Rotavirus	PRE	3615	88	2.43	χ^2^ test	<0.001	***
		POST	4700	221	4.70			
	Adenovirus	PRE	3406	204	5.99	χ^2^ test	<0.001	***
		POST	4700	121	2.57			

Note: Pathogen-specific detection rate was defined as the proportion of positive results for each individual shared target relative to the total number of diagnostic tests in which that target was actively investigated within the corresponding study period. Comparisons between pre- and post-implementation periods were performed using the χ^2^ test or Fisher’s exact test, as appropriate based on expected cell counts. Counts refer to aggregated detection events derived from test-level data and do not correspond to unique patients or stool samples. Because individual specimen-level linkage was not available, co-detections within the same sample could not be identified or adjusted for. In the post-implementation period, shared targets were systematically assessed as part of multiplex syndromic panels, whereas in the pre-implementation period, testing was performed selectively according to conventional methods and clinical suspicion; consequently, denominators may differ between targets and study periods. Norovirus detections in the post-implementation period include both GI and GII genogroups, aggregated at genus level for comparability with the pre-implementation period. Statistical significance levels are reported as *** *p* < 0.001.

**Table 4:** Diagnostic workload and turnaround time before and after implementation of multiplex syndromic panels.

Indicator	Pre-Implementation (Jan 2022–Jul 2023)	Post-Implementation (Jan 2024–Jul 2025)	Before–After Change (%)
Total diagnostic tests, *n*	25,574	24,509	−4.2%
Bacterial tests, *n*	15,987	19,809	+23.9%
Viral tests, *n*	9587	4700	−51%
Mean TAT from sampling, h	63	47	−25.4%
Mean TAT from laboratory check-in, h	56	41	−26.8%

Note: Data represent aggregated counts of diagnostic tests performed and aggregated turnaround time (TAT) estimates calculated at study-period level. Because individual specimen-level timestamps and variability measures were not available, no inferential statistical testing was performed for TAT comparisons.

**Table 5:** Summary of key economic and operational indicators before and after implementation of multiplex syndromic panels.

Metric	Pre-Implementation	Post-Implementation	Absolute Change	Relative Change (%)
Direct diagnostic costs (€)	324,538.90	343,126.00	+18,587.10	+5.7
Personnel costs (€)	249,375.00	154,375.00	−95,000.00	−38.1
Total laboratory costs (€)	573,913.90	497,501.00	−76,412.90	−13.3
Total pathogen detection events (*n*)	1388	2839	+1451	+104.5
Cost per diagnostic detection (€)	233.80	120.86	−112.94	−48.3
Mean TAT from sample collection (h)	63	47	−16	−25.4
Mean TAT from laboratory check-in (h)	56	41	−15	−26.8
Economic impact per hour of TAT reduction, overall laboratory costs (€) *	—	—	−4775.81 (sampling)/−5094.19 (check-in)	—
Economic impact per hour of TAT reduction, direct diagnostic costs only (€) **	—	—	+1161.69 (sampling)/+1239.14 (check-in)	—

Note: Direct diagnostic costs include instrument-related costs and reagents/consumables. Total laboratory costs include direct diagnostic costs plus personnel costs. Total pathogen detection events refer to aggregated pathogen detection events and do not correspond to unique stool samples or individual patients. * Negative values indicate net savings associated with shorter turnaround time when overall laboratory costs were considered. ** Positive values indicate additional expenditure associated with shorter turnaround time when only direct diagnostic costs were considered. Detailed costing by diagnostic pathway is provided in Supplementary Tables S16–S22.

## Data Availability

The original contributions presented in this study are included in the article/Supplementary Materials. Further inquiries can be directed to the corresponding author.

## Supplementary Materials

The following supporting information can be downloaded at https://www.mdpi.com/article/10.3390/microorganisms14071559/s1: Table S1: Costing framework and formulas used in the cost–consequence analysis; Table S2: Comparison of diagnostic targets before and after implementation of multiplex syndromic panels; Table S3: Diagnostic activity by pathogen category and healthcare setting in the pre- and post-implementation periods; Table S4: Change in testing volume for shared viral pathogens before and after implementation of multiplex syndromic panels (PRE vs. POST); Table S5: Diagnostic pathogen detection using conventional methods in the pre-implementation period (January 2022–July 2023); Table S6: Absolute detection of enteric pathogens in the pre-implementation period, stratified by age group; Table S7: Diagnostic pathogen detection using multiplex syndromic panels in the post-implementation period (January 2024–July 2025); Table S8A: Absolute detection of bacterial pathogens after implementation of multiplex syndromic panels, stratified by age group (POST); Table S8B: Absolute detection of viral pathogens after implementation of multiplex syndromic panels, stratified by age group (POST); Table S8C: Absolute detection of pathogens after implementation of multiplex syndromic panels, stratified by healthcare setting (POST); Table S9: Absolute detection of shared enteric pathogens before and after implementation of multiplex syndromic panels (PRE vs. POST); Table S10: Overall and category-specific test positivity by healthcare setting before and after implementation of multiplex syndromic panels; Table S11: Bacterial test positivity by age group and healthcare setting before and after implementation of multiplex syndromic panels; Table S12: Viral test positivity by age group and healthcare setting before and after implementation of multiplex syndromic panels; Table S13: Pathogen-specific detection rates for shared enteric targets by healthcare setting (PRE vs. POST); Table S14: Pathogen-specific detection rates for shared enteric targets by age group before and after implementation; Table S15: Organisational comparison between pre- and post-implementation workflows; Table S16: Estimated cost of conventional diagnostics in the pre-implementation period; Table S17: Estimated cost of syndromic diagnostics in the post-implementation period; Table S18: Estimated personnel costs in the pre- and post-implementation periods; Table S19: Cost per diagnostic detection in the pre- and post-implementation periods; Table S20: Cost composition analysis in the pre- and post-implementation periods; Table S21: Economic efficiency relative to turnaround time reduction; Table S22: Economic efficiency relative to turnaround time reduction based on direct diagnostic costs only; Figure S1: Category-specific test positivity before (PRE) and after (POST) implementation of multiplex syndromic panels. Bacterial test positivity increased from 4.47% to 8.91%, and viral test positivity increased from 7.03% to 22.85% after implementation of syndromic testing (χ^2^ test, *p* < 0.001 for both comparisons); Figure S2: Test positivity by healthcare setting before (PRE) and after (POST) implementation of multiplex syndromic panels. Bacterial test positivity increased in both community-based (4.0% to 8.5%) and hospitalised patients (5.5% to 9.8%). Viral test positivity also increased markedly in community-based (7.54% to 23.73%) and hospitalised patients (6.52% to 21.56%) (χ^2^ test, *p* < 0.001 for all comparisons); Figure S3: Bacterial diagnostic yield by age group and healthcare setting before (PRE) and after (POST) implementation of multiplex syndromic panels. Bacterial test positivity increased significantly in both age groups across community-based (≤14 years: 7.6% to 13.5%; >14 years: 3.0% to 7.3%) and hospital settings (≤14 years: 6.4% to 9.2%; >14 years: 5.1% to 10.0%) (χ^2^ test, *p* < 0.001 for all comparisons); Figure S4: Pathogen-specific detection rates for shared bacterial targets before (PRE) and after (POST) implementation of multiplex syndromic panels. Detection rates increased for *Campylobacter* spp. (3.16% to 4.22%) and *Shigella* spp. (0.01% to 0.30%), while remaining numerically stable for *Salmonella* spp. (1.61% to 1.50%) and Yersinia enterocolitica (0.37% to 0.34%). *Vibrio* spp. were detected only in the POST period (0.03%) (χ^2^ or Fisher’s exact test, *p* < 0.001 where applicable); Figure S5: Pathogen-specific detection rates for shared viral targets before (PRE) and after (POST) implementation of multiplex syndromic panels. Norovirus detection rate decreased from 14.89% to 10.00%, and Adenovirus from 5.99% to 2.57%, whereas Rotavirus increased from 2.43% to 4.70% after implementation (χ^2^ test, *p* < 0.001 for all comparisons); Figure S6: Pathogen-specific detection rates for shared bacterial targets by healthcare setting before (PRE) and after (POST) implementation of multiplex syndromic panels. In the community setting, detection rates increased for *Campylobacter* spp. (3.00% to 4.33%) and *Shigella* spp. (0.01% to 0.35%), while remaining stable for *Salmonella* spp., Yersinia enterocolitica, and *Vibrio* spp. Among hospitalised patients, *Shigella* spp. increased (0.00% to 0.20%), whereas other targets showed no significant change (χ^2^ or Fisher’s exact test, *p* < 0.001 where applicable); Figure S7: Pathogen-specific detection rates for shared viral targets by healthcare setting before (PRE) and after (POST) implementation of multiplex syndromic panels. In the community setting, Norovirus and Adenovirus detection rates decreased (18.34% to 10.51% and 5.54% to 2.43%, respectively), while Rotavirus increased (1.21% to 3.04%) (*p* < 0.001). Among hospitalised patients, Rotavirus increased (3.67% to 7.15%) and Adenovirus decreased (6.39% to 2.79%) (*p* < 0.001), whereas Norovirus showed no significant change (10.97% to 9.25%; ns); Figure S8: Pathogen-specific detection rates for shared bacterial targets by age group before (PRE) and after (POST) implementation of multiplex syndromic panels. Among patients aged ≤ 14 years, detection rates increased for *Campylobacter* spp. (3.83% to 5.51%), *Shigella* spp. (0.00% to 0.30%), and Yersinia enterocolitica (0.00% to 0.65%), while *Salmonella* spp. remained stable. In patients aged > 14 years, *Campylobacter* spp. (2.96% to 3.89%) and *Shigella* spp. (0.01% to 0.30%) increased, whereas *Salmonella* spp., Yersinia enterocolitica, and *Vibrio* spp. showed no significant change (χ^2^ or Fisher’s exact test, *p* < 0.05 where applicable); Figure S9: Pathogen-specific detection rates for shared viral targets by age group before (PRE) and after (POST) implementation of multiplex syndromic panels. In children aged ≤6 years, Norovirus decreased (19.64% to 13.00%) and Rotavirus increased (2.61% to 7.24%), while Adenovirus remained stable (4.90% to 5.10%). In individuals aged > 6 years, Adenovirus decreased markedly (7.24% to 0.54%), whereas Norovirus and Rotavirus showed no significant change (χ^2^ test, *p* < 0.001 where applicable); Figure S10: Diagnostic workload and turnaround time before (PRE) and after (POST) implementation of multiplex syndromic panels. Supplementary Materials are available in the following file: Supplementary Material: Diagnostic and Microbiological Impact of Multiplex Syndromic Testing for Acute Infectious Gastroenteritis in a Regional Laboratory Network—A Real-World Before–After Study.

## Abbreviations

The following abbreviations are used in this manuscript:

AIG	Acute infectious gastroenteritis
ARAN	Agenzia per la Rappresentanza Negoziale delle Pubbliche Amministrazioni
CET-CEROM	Comitato Etico Territoriale della Romagna
CIN	cefsulodin–irgasan–novobiocin agar
COS	Columbia blood agar
EIEC	enteroinvasive *Escherichia coli*
EMR	electronic medical record
EU GDPR	European Union General Data Protection Regulation
GI	gastrointestinal
GI/GII	genogroup I/genogroup II
LIS	laboratory information system
MALDI-TOF MS	matrix-assisted laser desorption/ionisation time-of-flight mass spectrometry
MCK	MacConkey agar
PCR	polymerase chain reaction
PRE	pre-implementation period
POST	post-implementation period
SS	Salmonella–Shigella agar
TAT	turnaround time
XLD	xylose lysine deoxycholate agar
