# Supplementary material for: Diagnostic and Microbiological Impact of Multiplex Syndromic Testing for Acute Infectious Gastroenteritis in a Regional Laboratory Network: A Real-World Before–After Study
Source: Microorganisms. 2026 Jul 16;14(7):1559. doi: 10.3390/microorganisms14071559 (PMC13413444; doi:10.3390/microorganisms14071559)
Supplement: Supplementary file 1 [file microorganisms-14-01559-s001.zip › microorganisms-4427370-supplementary.pdf]

Supplementary material

Table

Table S1 *Costing framework and formulas used in the cost-consequence analysis.*

Component	Formula
Conventional bacterial diagnostics	$C_{tot,PRE\text{-}bacterial} = (N_{copro} \times C_{copro}) + (N_{copro\ pos} \times C_{MALDI}) + (N_{yer\ neg} \times C_{yer\ neg}) + (N_{yer\ pos} \times C_{yer\ pos}) + (N_{vib\ neg} \times C_{vib\ neg}) + (N_{vib\ pos} \times C_{vib\ pos})$
Routine stool culture	$C_{copro} = C_{instrument} + C_{culture\ media/enrichment\ broth}$
MALDI-TOF MS identification	$N_{copro\ pos} \times C_{MALDI}$
Yersinia spp. negative testing	$C_{yer\ neg} = C_{instrument} + C_{Yersinia\ medium}$
Yersinia spp. positive testing	$C_{yer\ pos} = C_{instrument} + C_{Yersinia\ medium} + C_{MALDI}$
Vibrio spp. negative testing	$C_{vib\ neg} = C_{manual\ culture\ media}$
Vibrio spp. positive testing	$C_{vibrio\ pos} = C_{manual\ culture\ media} + C_{MALDI}$
Conventional viral diagnostics	$C_{tot,PRE\text{-}viral} = (N_{noro} \times C_{noro}) + (N_{rota} \times C_{rota}) + (N_{adeno} \times C_{adeno})$
Norovirus testing	$C_{noro} = C_{instrument,GeneXpert} + C_{Norovirus\ reagents/consumables}$
Rotavirus testing	$C_{rota} = C_{instrument,LIAISON\ XL} + C_{Rotavirus\ reagents/consumables}$
Adenovirus testing	$C_{adeno} = C_{instrument,LIAISON\ XL} + C_{Adenovirus\ reagents/consumables}$
Syndromic molecular diagnostics	$C_{tot,POST} = (N_{bact} \times C_{bact\ panel}) + (N_{viral} \times C_{viral\ panel})$
Bacterial syndromic panel	$C_{bact\ panel} = C_{instrument} + C_{bacterial\ panel\ reagents/consumables}$

Viral syndromic panel	$C_{viral\ panel} = C_{instrument} + C_{viral\ panel\ reagents/consumables}$
Personnel costs	$C_{personnel} = (C_{technician} \times 19/12 \times 0.75) + (C_{biologist} \times 19/12 \times 0.75)$
Cost per diagnostic detection, pre-implementation	$C_{per\ detection,PRE} = (C_{tot,PRE\text{-}bacterial} + C_{tot,PRE\text{-}viral}) / (N_{detection,PRE\text{-}bacterial} + N_{detection,PRE\text{-}viral})$
Cost per diagnostic detection, post-implementation	$C_{per\ detection,POST} = (C_{tot,POST\text{-}bacterial} + C_{tot,POST\text{-}viral}) / (N_{detection,POST\text{-}bacterial} + N_{detection,POST\text{-}viral})$
Economic efficiency relative to TAT reduction	Cost per hour saved = $(C_{tot,POST} - C_{tot,PRE}) / (TAT_{PRE} - TAT_{POST})$

Abbreviations: *C, cost; PRE, pre-implementation period; POST, post-implementation period; N, number of tests or detections; TAT, turnaround time; MALDI-TOF MS, matrix-assisted laser desorption/ionisation time-of-flight mass spectrometry.*

Table S2 *Comparison of diagnostic targets before and after implementation of multiplex syndromic panels*

Pathogen category	Pathogen	Pre-implementation (conventional workflow)	Post-implementation (syndromic panels)
**Bacteria**	*Salmonella spp*	✓ (culture)	✓ Allplex™ GI-Bacteria(I) Assay
	Campylobacter spp.	✓ (culture)	✓ Allplex™ GI-Bacteria(I) Assay
	Shigella spp.	✓ (culture)	✓ Allplex™ GI-Bacteria(I) Assay
	Aeromonas spp.	✕	✓ Allplex™ GI-Bacteria(I) Assay
	Shigella spp./EIEC	✕	✓ Allplex™ GI-Bacteria(I) Assay
	Yersinia enterocolitica	✓ (culture)	✓ Allplex™ GI-Bacteria(I) Assay
	Vibrio spp.	✓ (culture)	✓ Allplex™ GI-Bacteria(I) Assay
**Viruses**	*Norovirus*	✓ Xpert® Norovirus assay	✓Allplex™ GI-Virus Assay

Rotavirus	✓ LIAISON® Rotavirus chemiluminescent immunoassays	✓Allplex™ GI-Virus Assay
Adenovirus	✓ LIAISON® Adenovirus chemiluminescent immunoassays	✓Allplex™ GI-Virus Assay
Astrovirus	✕	✓Allplex™ GI-Virus Assay
Sapovirus	✕	✓Allplex™ GI-Virus Assay
Norovirus GII	✕	✓Allplex™ GI-Virus Assay

Note: *In the pre-implementation period, pathogen detection was based on selective culture-based methods and single-target viral assays requested according to clinical suspicion. In the post-implementation period, multiplex syndromic panels enabled the simultaneous detection of all listed targets from a single stool sample.*

Table S3 *Diagnostic activity by pathogen category and healthcare setting in the pre- and post-implementation periods*

Pathogen category	Study period	Healthcare setting	Bacterial tests performed, n	Proportion of total (%)	Relative change vs pre (%)
		Community (outpatients / ED)	10,987	68.7	-
**Bacterial**	**Pre-implementation**	Hospitalised patients	5,000	31.3	-
		**Total**	**15,987**	**100**	-
		Community (outpatients / ED)	13,447	67.9	+22.4
**Bacterial**	**Post-implementation**	Hospitalised patients	6,362	32.1	+27.2
		**Total**	**19,809**	**100**	**+23.9**
		Community (outpatients / ED)	4,785	49.9	-
**Viral**	**Pre-implementation**				
		Hospitalised patients	4,802	50.1	-

		**Total**	**9,587**	**100**	-
		Community (outpatients / ED)	2,798	59.5	-41.5%
**Viral**	**Post-implementation**	Hospitalised patients	1,902	40.5	-60.4%
		**Total**	**4,700**	**100**	**-51.0%**

Note: *Diagnostic activity is reported as aggregated counts of tests performed and does not correspond to the number of unique stool samples analysed. Bacterial diagnostic activity includes culture-based assays in the pre-implementation period and multiplex molecular bacterial panels in the post-implementation period. Viral diagnostic activity includes single-target antigen-based or molecular assays in the pre-implementation period and multiplex molecular viral panels in the post-implementation period. Relative changes were calculated by comparing post-implementation and pre-implementation volumes within each healthcare setting.*

Table S4. *Change in testing volume for shared viral pathogens before and after implementation of multiplex syndromic panels (PRE vs POST)*

Pathogen	Setting	PRE (n)	POST (n)	Absolute change	Percentage change
**Norovirus**	Community (outpatients / ED)	1,363	2,798	+1,435	+105.3%
	Hospitalised patients	1,203	1,902	+699	+58.1%
	**Total**	**2,566**	**4,700**	**+2,134**	**+83.2%**
**Adenovirus**	Community (outpatients / ED)	1,607	2,798	+1,191	+74.1%
	Hospitalised patients	1,799	1,902	+103	+5.7%
	**Total**	**3,406**	**4,700**	**+1,294**	**+38.0%**
**Rotavirus**	Community (outpatients / ED)	1,815	2,797	+983	+54.2%
	Hospitalised patients	1,800	1,903	+103	+5.7%
	**Total**	**3,615**	**4,700**	**+1,086**	**+30.0%**

Note: *Data are reported as aggregated numbers of stool samples tested for each viral pathogen during the pre-implementation (single-target assays) and post-implementation (multiplex syndromic panels) periods, stratified by healthcare setting. Percentage change was calculated as (POST − PRE) / PRE × 100. Counts refer to tests performed and do not correspond to unique stool samples or individual patients. In the post-implementation period, all viral targets were tested systematically for each sample as part of the syndromic panel, whereas in the pre-implementation period testing was performed selectively based on clinical suspicion.*

Table S5. *Diagnostic pathogen detection using conventional methods in the pre-implementation period (Jan 2022–Jul 2023)*

Pathogen category	Pathogen	Detection method (PRE)	Positive detections, n
**Bacteria**	*Salmonella* spp.	Coproculture	239
	Campylobacter spp.	Coproculture	470
	Shigella spp.	Coproculture	1
	Yersinia enterocolitica	Selective culture (on request)	4
	Vibrio cholerae	Selective culture (on request)	0
	**Total bacterial detections**		**714**
**Viruses**	Norovirus	Single-target PCR	382
	Adenovirus	Antigen test	204
	Rotavirus	Antigen test	88
	**Total viral detections**		**674**

Note: *Data are reported as aggregated numbers of positive detections obtained during the pre-implementation period using conventional diagnostic methods (community + hospitalised patients). Counts refer to detected pathogens and do not correspond to unique stool samples or individual patients; co-detections may occur. Targets not listed were not routinely investigated.*

Table S6. *Absolute detection of enteric pathogens in the pre-implementation period, stratified by age group*

Pathogen category	Pathogen	Age group definition	Younger group, n	Older group, n	Total detections, n
Bacteria	*Salmonella* spp.	≤14 vs >14 years	132	107	239
	Campylobacter spp.	≤14 vs >14 years	134	336	470
	Shigella spp.	≤14 vs >14 years	0	1	1
	Yersinia enterocolitica	≤14 vs >14 years	0	4	4
	Vibrio cholerae	≤14 vs >14 years	0	0	0
	**Total bacterial detections**	≤14 vs >14 years	**266**	**448**	**714**
Viruses	Norovirus	≤6 vs >6 years	274	108	382
	Adenovirus	≤6 vs >6 years	162	42	204
	Rotavirus	≤6 vs >6 years	49	39	88
	**Total viral detections**	≤6 vs >6 years	**485**	**189**	**674**

Note: *Data are reported as absolute numbers of pathogen detections identified during the pre-implementation period using conventional diagnostic workflows. Bacterial pathogens were detected by standard coproculture and, in selected cases, by targeted culture-based assays for Yersinia enterocolitica and Vibrio cholerae based on clinical suspicion. Viral pathogens were detected using single-target diagnostic assays requested according to clinical suspicion, including molecular detection of Norovirus RNA and antigen-based assays for Rotavirus and Adenovirus. Age groups were defined a priori as ≤14 vs >14 years for bacterial pathogens and ≤6 vs >6 years for viral pathogens. Counts refer to pathogen detections and do not correspond to unique stool samples or individual patients; multiple detections may originate from the same sample. Negative results are not shown.*

Table S7. *Diagnostic pathogen detection using multiplex syndromic panels in the post-implementation period (Jan 2024–Jul 2025)*

**Pathogen category**	**Pathogen**	**GI-Panel**	**Positive detections, n**
Bacteria	*Aeromonas* spp.	Allplex™ GI-Bacteria(I) Assay	499
	Campylobacter spp.	Allplex™ GI-Bacteria(I) Assay	835
	Salmonella spp	Allplex™ GI-Bacteria(I) Assay	297
	Shigella spp./EIEC	Allplex™ GI-Bacteria(I) Assay	60
	Yersinia enterocolitica	Allplex™ GI-Bacteria(I) Assay	68
	Vibrio spp.	Allplex™ GI-Bacteria(I) Assay	6
	Total bacterial detections		**1765**
Viruses	Norovirus GI	Allplex™ GI-Virus Assay	70
	Norovirus GII	Allplex™ GI-Virus Assay	400
	Rotavirus	Allplex™ GI-Virus Assay	221
	Adenovirus	Allplex™ GI-Virus Assay	121
	Astrovirus	Allplex™ GI-Virus Assay	96
	Sapovirus	Allplex™ GI-Virus Assay	166
	Total viral detections		**1074**

Note: *Data are reported as aggregated numbers of positive detections identified during the post-implementation period using multiplex molecular syndromic panels. Bacterial pathogens were detected using the Allplex™ GI-Bacteria(I) Assay, and viral pathogens were detected using the Allplex™ GI-Virus Assay. Counts refer to pathogen detections and do not correspond to unique stool samples or individual patients; co-detections of multiple pathogens within the same sample are possible. Targets not listed were not routinely investigated in the present analysis.*

Table S8A. *Absolute detection of bacterial pathogens after implementation of multiplex syndromic panels, stratified by age group (POST)*

Pathogen	≤14 years, n	>14 years, n	Total detections, n
Aeromonas spp.	80	419	499
Campylobacter spp.	219	616	835
Salmonella spp	142	155	297
Shigella spp./	12	48	60
Yersinia enterocolitica	26	42	68
Vibrio spp.	0	6	6
**Total bacterial detections**	**479**	**1286**	**1765**

Note: *Data are reported as absolute numbers of positive detections for each bacterial pathogen identified during the post-implementation period using multiplex syndromic panels. Results are stratified by age group according to the predefined study categories. Counts refer to pathogen detections and not to unique stool samples or individual patients; multiple pathogens may be detected in the same sample. Negative results are not shown in this table, as the aim is to describe the absolute spectrum of bacterial pathogens detected following implementation of syndromic testing.*

Table S8B. *Absolute detection of viral pathogens after implementation of multiplex syndromic panels, stratified by age group (POST)*

Pathogen	≤6 years, n	>6 years, n	Total detections, n
Norovirus GI	37	33	70
Norovirus GII	236	164	400
Rotavirus	152	69	221
Adenovirus	107	14	121
Astrovirus	68	28	96

Sapovirus	134	32	166
**Total viral detections**	**734**	**340**	**1074**

Note: *Data represent absolute numbers of viral pathogen detections identified during the post-implementation period using multiplex syndromic panels, stratified by age group. Values correspond to positive detections and do not represent the number of unique stool samples or patients. Co-detections of multiple viral targets within the same sample are possible. Negative results are not included, as this table focuses on the descriptive distribution of viral pathogens detected after implementation of syndromic testing.*

Table S8C. *Absolute detection of pathogens after implementation of multiplex syndromic panels, stratified by healthcare setting (POST)*

Pathogen category	Community (outpatients / ED), n	Hospitalised patients, n	Total detections, n
Bacterial pathogens	1141	624	1765
Viral pathogens	664	410	1074
Overall detections	1805	1034	2839

Note: *Absolute numbers of bacterial and viral pathogen detections identified during the post-implementation period are reported according to healthcare setting (community vs hospitalised patients). Data refer to positive detections generated by multiplex syndromic panels and do not correspond to unique samples or patients. Multiple pathogens may be detected in a single specimen. Negative results are not shown, as the purpose of this table is to describe the distribution of detected pathogens across care settings rather than diagnostic yield.*

Table S9. *Absolute detection of shared enteric pathogens before and after implementation of multiplex syndromic panels (PRE vs POST)*

Pathogen category	Pathogen	PRE, n	POST, n	Absolute difference,n
Bacteria	*Salmonella* spp.	239	297	+58
	Campylobacter spp.	470	835	+365
	Shigella spp.	1	60	+59
	Yersinia enterocolitica	4	68	+64
	Vibrio spp./*V. cholerae*	0	6	+6
	**Total bacterial detections (shared targets)**	**714**	**1,266**	**+552**

Viruses	Norovirus	382	470*	+88
	Rotavirus	88	221	+133
	Adenovirus	204	121	-83
	**Total viral detections (shared targets)**	**674**	**812**	**+138**

Note: *Norovirus detections in the POST period include both GI and GII genogroups; for comparability with the PRE period, counts are aggregated at the genus level. Data are reported as absolute numbers of pathogen detections for targets investigated in both study periods. In the pre-implementation period, pathogen detection was based on selective culture-based methods and single-target viral assays requested according to clinical suspicion. In the post-implementation period, detections were generated by multiplex molecular syndromic panels. Counts refer to pathogen detections and do not correspond to unique stool samples or individual patients; co-detections of multiple pathogens within the same sample are possible. Differences between periods reflect changes in diagnostic strategy and testing coverage rather than inferred changes in disease incidence.*

Table S10. *Overall and category-specific test positivity by healthcare setting before and after implementation of multiplex syndromic panels*

Analysis level	Healthcare setting	Study period	Tests performed, n	Positive detections, n	Positivity rate (%)	Statistical test	p value	Significance
Overall	Community (outpatients / ED)	PRE	15,772	801	5.08	χ^2 test	<0.001	***
		POST	16,245	1,805	11.11			
	Hospitalised patients	PRE	9,802	587	5.99	χ^2 test	<0.001	***
		POST	8,264	1,034	12.51			
Bacterial pathogens	Community (outpatients / ED)	PRE	10,987	440	4.00	χ^2 test	<0.001	***
		POST	13,447	1,141	8.50			
	Hospitalised patients	PRE	5,000	274	5.50	χ^2 test	<0.001	***
		POST	6,362	624	9.80			
Viral pathogens	Community (outpatients / ED)	PRE	4,785	361	7.54	χ^2 test	<0.001	***

	POST	2,798	664	23.73			
Hospitalised patients	PRE	4,802	313	6.52	χ² test	<0.001	***
	POST	1,902	410	21.56			

Note: *Test positivity was defined as the proportion of diagnostic tests yielding at least one pathogen detection within each healthcare setting, study period, or pathogen category. Comparisons between pre- and post-implementation periods were performed using the χ^2 test for independence. Counts refer to aggregated test-level detection events and do not correspond to unique stool samples or individual patients. In the post-implementation period, all targets within each diagnostic category were systematically assessed as part of multiplex syndromic panels, whereas in the pre-implementation period testing was performed selectively according to conventional methods and clinical suspicion. Statistical significance levels are reported as *** p < 0.001; ** p < 0.01; * p < 0.05; ns, not significant.*

Table S11. *Bacterial test positivity by age group and healthcare setting before and after implementation of multiplex syndromic panels*

Analysis level	Healthcare setting	Age group	Study period	Bacterial tests, n	Positive detections, n	Positivity rate (%)	Statistical test	p value	Significance
Overall	All settings	≤14 years*	PRE	3,690	266	7.21	χ² test	<0.001	***
			POST	3,987	479	12.02			
Overall	All settings	>14 years*	PRE	12,297	448	3.64	χ² test	<0.001	***
			POST	15,831	1,286	8.12			
Stratified	Community (outpatients / ED)	≤14 years*	PRE	2,402	183	7.62	χ² test	<0.001	***
			POST	2,637	355	13.46			
Stratified	Community (outpatients / ED)	>14 years*	PRE	8,585	257	2.99	χ² test	<0.001	***
			POST	10,810	786	7.27			
Stratified	Hospitalised patients	≤14 years*	PRE	1,288	83	6.44	χ² test	<0.001	***

			POST	1,341	124	9.25			
Stratified	Hospitalised patients	>14 years*	PRE	3,712	191	5.14	χ^2 test	<0.001	***
			POST	5,021	500	9.96			

Note: *Test positivity was defined as the proportion of bacterial diagnostic tests yielding at least one bacterial pathogen detection within each age group, healthcare setting, and study period. Comparisons between pre- and post-implementation periods were performed using the χ^2 test for independence. Counts refer to aggregated test-level detection events and do not correspond to unique stool samples or individual patients. Due to the aggregated structure of the dataset, co-detections within the same specimen could not be individually reconstructed. Age groups were defined a priori according to clinical and epidemiological criteria. Statistical significance levels are reported as *** p < 0.001; ** p < 0.01; * p < 0.05; ns, not significant.*

Table S12. *Viral test positivity by age group and healthcare setting before and after implementation of multiplex syndromic panels*

Analysis level	Healthcare setting	Age group	Study period	Viral tests, n	Positive detections, n	Positivity rate (%)	Statistical test	p value	Significance
Overall	All settings	≤6 years	PRE	5,092	485	9.53	χ^2 test	<0.001	***
			POST	2,100	734	34.95			
Overall	All settings	>6 years	PRE	4,495	189	4.20	χ^2 test	<0.001	***
			POST	2,600	340	13.08			
Stratified	Community (outpatients / ED)	≤6 years	PRE	2,450	255	10.41	χ^2 test	<0.001	***
			POST	1,208	460	38.08			
Stratified	Community (outpatients / ED)	>6 years	PRE	2,335	106	4.54	χ^2 test	<0.001	***
			POST	1,590	204	12.83			
Stratified	Hospitalised patients	≤6 years	PRE	2,642	230	8.71	χ^2 test	<0.001	***
			POST	892	274	30.72			

Stratified	Hospitalised patients	>6 years	PRE	2,160	83	3.84	χ^2 test	<0.001	***
			POST	1,010	136	13.47			

Note: *Test positivity was defined as the proportion of viral diagnostic tests yielding at least one viral pathogen detection within each age group, healthcare setting, and study period. Comparisons between pre- and post-implementation periods were performed using the χ^2 test for independence. Counts refer to aggregated test-level detection events and do not correspond to unique stool samples or individual patients. Due to the aggregated structure of the dataset, co-detections within the same specimen could not be individually reconstructed. Age groups were defined a priori according to clinical and epidemiological criteria. Statistical significance levels are reported as *** $p < 0.001$; ** $p < 0.01$; * $p < 0.05$; ns, not significant.*

Table S13*. Pathogen-specific detection rates for shared enteric targets by healthcare setting (PRE vs POST)*

Pathogen category	Pathogen	Healthcare setting	Study period	Tests performed, n	Positive detections, n	Positivity rate (%)	Statistical test	p value	Significance
Bacterial	Salmonella spp.	Community	PRE	9,865	139	1.41	χ^2 test	0.31	ns
			POST	13,447	166	1.23			
	Campylobacter spp.	Community	PRE	9,865	296	3.00	χ^2 test	<0.001	***
			POST	13,447	582	4.33			
	Shigella spp.	Community	PRE	9,865	1	0.01	Fisher's exact	<0.001	***
			POST	13,447	47	0.35			
	Yersinia enterocolitica	Community	PRE	1,068	4	0.37	Fisher's exact	0.69	ns
			POST	13,447	56	0.42			
	Vibrio spp.	Community	PRE	54	0	0.00	Fisher's exact	1.00	ns
			POST	13,447	3	0.02			
	Salmonella spp.	Hospitalised patients	PRE	5,000	100	2.00	χ^2 test	0.84	ns

			POST	6,362	131	2.06			
	Campylobacter spp.	Hospitalised patients	PRE	5,000	174	3.48	χ^2 test	0.18	ns
			POST	6,362	253	3.98			
	Shigella spp.	Hospitalised patients	PRE	5,000	0	0.00	Fisher's exact	0.001	**
			POST	6,362	13	0.20			
	Yersinia enterocolitica	Hospitalised patients	PRE	0	0	—	Not testable	Not testable	Not testable
			POST	6,362	12	0.19			
	Vibrio spp.	Hospitalised patients	PRE	0	0	—	Not testable	Not testable	Not testable
			POST	6,362	3	0.05			
Viral	Norovirus	Community	PRE	1,363	250	18.34	χ^2 test	<0.001	***
			POST	2,798	294	10.51			
	Rotavirus	Community	PRE	1,815	22	1.21	χ^2 test	<0.001	***
			POST	2,798	85	3.04			
	Adenovirus	Community	PRE	1,607	89	5.54	χ^2 test	<0.001	***
			POST	2,798	68	2.43			
	Norovirus	Hospitalised patients	PRE	1,203	132	10.97	χ^2 test	0.11	ns
			POST	1,902	176	9.25			

	Rotavirus	Hospitalised patients	PRE	1,800	66	3.67	χ^2 test	<0.001	***
			POST	1,902	136	7.15			
	Adenovirus	Hospitalised patients	PRE	1,799	115	6.39	χ^2 test	<0.001	***
			POST	1,902	53	2.79			

Note: *Pathogen-specific detection rate was defined as the proportion of positive results for each individual shared target relative to the total number of diagnostic tests in which that target was actively investigated within the corresponding healthcare setting and study period. Comparisons between pre- and post-implementation periods were performed using the χ^2 test or Fisher's exact test, as appropriate based on expected cell counts. Counts refer to aggregated detection events derived from test-level data and do not correspond to unique patients or stool samples. Because individual specimen-level linkage was not available, co-detections within the same sample could not be identified or adjusted for. In the post-implementation period, all shared targets were systematically assessed as part of multiplex syndromic panels, whereas in the pre-implementation period testing was performed selectively according to conventional methods and clinical suspicion; consequently, denominators may differ between targets, healthcare settings, and study periods. When no tests were performed in the pre-implementation period, statistical comparison was not applicable. Statistical significance levels are reported as *** p < 0.001; ** p < 0.01; * p < 0.05; ns, not significant.*

Table S14. *Pathogen-specific detection rates for shared enteric targets by age group before and after implementation*

Pathogen category	Pathogen	Age group*	Study period	Tests performed, n	Positive detections, n	Positivity rate (%)	Statistical test	p value	Significance
Bacterial	Salmonella spp.	≤14 years	PRE	3,502	132	3.77	χ^2 test	0.67	ns
			POST	3,978	142	3.57			
	Campylobacter spp.	>14 years	PRE	11,363	107	0.94	χ^2 test	0.73	ns
			POST	15,831	155	0.98			
	Shigella spp.	≤14 years	PRE	3,502	134	3.83	χ^2 test	<0.001	***
			POST	3,978	219	5.51			
	Yersinia enterocolitica	>14 years	PRE	11,363	336	2.96	χ^2 test	<0.001	***
			POST	15,831	616	3.89			

	Vibrio spp.	≤14 years	PRE	3,502	0	0.00	Fisher's exact	0.002	**
			POST	3,978	12	0.30			
	Salmonella spp.	>14 years	PRE	11,363	1	0.01	Fisher's exact	<0.001	***
			POST	15,831	48	0.30			
	Campylobacter spp.	≤14 years	PRE	186	0	0.00	Fisher's exact	0.04	*
			POST	3,978	26	0.65			
	Shigella spp.	>14 years	PRE	882	4	0.45	χ^2 test	0.21	ns
			POST	15,831	42	0.27			
	Yersinia enterocolitica	≤14 years	PRE	2	0	0.00	Not testable	NA	NA
			POST	3,978	0	0.00			
	Vibrio spp.	>14 years	PRE	52	0	0.00	Fisher's exact	0.62	ns
			POST	15,831	6	0.04			
	Norovirus	≤6 years	PRE	1,395	274	19.64	χ^2 test	<0.001	***
			POST	2,100	273	13.00			
Viral	Rotavirus	>6 years	PRE	1,171	108	9.22	χ^2 test	0.07	ns
			POST	2,600	197	7.58			
	Adenovirus	≤6 years	PRE	1,879	49	2.61	χ^2 test	<0.001	***

		POST	2,100	152	7.24			
Norovirus	>6 years	PRE	1,736	39	2.25	χ^2 test	0.39	ns
		POST	2,600	69	2.65			
Rotavirus	≤6 years	PRE	1,818	89	4.90	χ^2 test	0.82	ns
		POST	2,100	107	5.10			
Adenovirus	>6 years	PRE	1,588	115	7.24	χ^2 test	<0.001	***
		POST	2,600	14	0.54			

Note: *Pathogen-specific detection rate was defined as the proportion of positive results for each individual shared target relative to the total number of diagnostic tests in which that target was actively investigated within the corresponding age group and study period. Comparisons between pre- and post-implementation periods were performed using the χ^2 test or Fisher's exact test, as appropriate based on expected cell counts. Counts refer to aggregated detection events derived from test-level data and do not correspond to unique patients or stool samples. Because individual specimen-level linkage was not available, co-detections within the same sample could not be identified or adjusted for. In the post-implementation period, all shared targets were systematically assessed as part of multiplex syndromic panels, whereas in the pre-implementation period testing was performed selectively according to conventional methods and clinical suspicion; consequently, denominators may differ between targets, age groups, and study periods. When no tests were performed in the pre-implementation period, statistical comparison was not applicable. Age groups were defined a priori as ≤14 vs >14 years for bacterial targets and ≤6 vs >6 years for viral targets. Statistical significance levels are reported as *** p < 0.001; ** p < 0.01; * p < 0.05; ns, not significant.*

Table S15. *Organisational comparison between pre- and post-implementation workflows*

Variable	Pre-implementation	Post-implementation
Workflow structure	Multi-step, heterogeneous	Integrated, standardised
Diagnostic approach	Culture + single-target tests	Multiplex syndromic panels
Viral diagnostics	Separate platforms (GeneXpert, LIAISON XL)	Integrated PCR panel
Bacterial diagnostics	Culture-based	Molecular
Number of platforms	Multiple	Single integrated system

Personnel required	3 technicians + 1 biologist	1 technician + 1 biologist
Manual handling	High	Low
Number of analytical steps	High	Reduced
Need for downstream identification	Yes (MALDI-TOF)	No
Laboratory space requirement	High	Reduced
Risk of manual error	Higher	Lower

Note: *Organisational variables were assessed descriptively by comparing conventional (pre-implementation) and syndromic (post-implementation) workflows, including differences in analytical steps, platforms, personnel requirements, and laboratory space utilisation. No inferential statistical analysis was performed.*

Table S16. *Estimated cost of conventional diagnostics in the pre-implementation period*

Diagnostic category	Diagnostic pathway	Number of tests (n)	Unit cost (€)	Total cost (€)	Share of total cost (%)
Bacterial	Routine stool culture	14,865	7.10	105,541.50	32.50
	MALDI-TOF MS identification (after positive stool culture)	710	2.50	1,775.00	0.50
	Yersinia spp. negative	1,064	3.90	4,149.60	1.30
	Yersinia spp. positive	4	6.40	25.60	<0.1
	Vibrio spp. negative	54	2.70	145.80	<0.1
	Vibrio spp. positive	0	5.20	0.00	0.00
	**Subtotal bacterial**			**111,637.50**	34.40
Viral	Norovirus RNA testing	2,566	47.40	121,628.40	37.50

	Adenovirus antigen testing	3,406	13.00	44,278.00	13.60
	Rotavirus antigen testing	3,615	13.00	46,995.00	14.50
	**Subtotal viral**			**212,901.40**	65.60
Total				324,538.90	100.00

Note: *Costs are reported as estimated direct laboratory costs for conventional diagnostics in the pre-implementation period. Bacterial costs include routine stool culture, MALDI-TOF MS identification of positive cultures, and targeted testing for Yersinia spp. and Vibrio spp. Viral costs include Norovirus RNA testing and Adenovirus/Rotavirus antigen testing. Percentages were calculated relative to the total estimated pre-implementation diagnostic cost..*

Table S17. *Estimated cost of syndromic diagnostics in the post-implementation period*

Diagnostic category	Diagnostic pathway	Number of tests (n)	Unit cost (€)	Total cost (€)	Share of total cost (%)
Bacterial	GI bacterial panel	19,809	14.00	277,326.00	80.80
Viral	GI viral panel	4,700	14.00	65,800.00	19.20
Total				343,126.00	100.00

Note: *costs are reported as estimated direct laboratory costs for syndromic diagnostics in the post-implementation period. Unit costs include instrument-related costs, assay-specific reagents, consumables, and nucleic acid extraction. Bacterial and viral costs refer to multiplex syndromic panels (Allplex™ GI-Bacteria(I) Assay and Allplex™ GI-Virus Assay, respectively). Percentages were calculated relative to the total estimated post-implementation diagnostic cost.*

Table S18*. Estimated personnel costs in the pre- and post-implementation periods*

Study period	Biomedical Laboratory Technicians (n)	Clinical Biologists (n)	Technician cost (19 mo, €)	Biologist cost (19 mo, €)	Total personnel cost (€)
Pre-implementation	3	1	47,500.00	106,875.00	249,375.00
Post-implementation	1	1	47,500.00	106,875.00	154,375.00
Absoluta change					−95,000.00

Relative change	−38.1%

Note: *costs were estimated according to the costing model described in the Methods section, using annual cost assumptions for one Biomedical Laboratory Technician (D3 category) and one Clinical Biologist, normalised to the 19-month study period and adjusted by a multiplicative factor of 0.75 to reflect the proportion of working time attributable to gastroenterological diagnostics. Annual cost assumptions were informed by the applicable national collective labour agreements and ARAN remuneration data.*

Table S19. *Cost per diagnostic detection in pre- and post-implementation periods*

Metric	Pre-implementation	Post-implementation
Total cost (€)	324,538.90	343,126.00
Total detections (n)	1,388	2,839
Cost per detection (€)	233.80	120.86
Absolute change (€)		−112.94
Relative change (%)		−48.3

Table S20. *Cost composition analysis in pre- and post-implementation periods*

Cost component	Pre-implementation(€)	Pre-implementation(%)	Post-implementation(€)	Post-implementation(%)	Absolute change(€)	Relative change (%)	Change in share (pp)
Instrument-related costs	113,369.40	19.80	49,018.00	9.90	−64,351.40	−56.8	−9.9
Reagents and consumables	211,169.50	36.80	294,108.00	59.10	+82,938.50	+39.3	+22.3
Personnel	249,375.00	43.40	154,375.00	31.00	−95,000.00	−38.1	−12.4
Total	573,913.90	100.00	497,501.00	100.00	−76,412.90	−13.3	—

Note: *Costs are reported as overall laboratory costs, including instrument-related costs, reagents/consumables, and personnel. Instrument-related costs were separated from total diagnostic costs to avoid double counting. Percentages were calculated relative to the total cost in each study period. Relative change was calculated as the percentage difference between post-implementation and pre-implementation values. Change in share was expressed as percentage-point difference between study periods.*

Table S21. *Economic efficiency relative to turnaround time reduction*

Metric	Pre-implementation	Post-implementation	Difference	Cost per hour saved (€)
Overall laboratory cost (€)	573,913.90	497,501.00	−76,412.90	—
Mean TAT from sampling (h)	63	47	−16	−4,775.81
Mean TAT from laboratory check-in (h)	56	41	−15	−5,094.19

Note: *Negative values indicate that the reduction in turnaround time was associated with a net reduction in overall laboratory costs. Overall laboratory costs included instrument-related costs, reagents/consumables, and personnel costs.*

Table S22. *Economic efficiency relative to turnaround time reduction based on direct diagnostic costs only*

Metric	Pre-implementation	Post-implementation	Difference	Cost per hour saved (€)
Direct diagnostic cost (€)	324,538.90	343,126.00	+18,587.10	—
Mean TAT from sampling (h)	63	47	−16	1,161.69
Mean TAT from laboratory check-in (h)	56	41	−15	1,239.14

Note: *Positive values indicate that the reduction in turnaround time was associated with an increase in direct diagnostic expenditure. Direct diagnostic costs included instrument-related costs and reagents/consumables, but excluded personnel costs.*

Figures

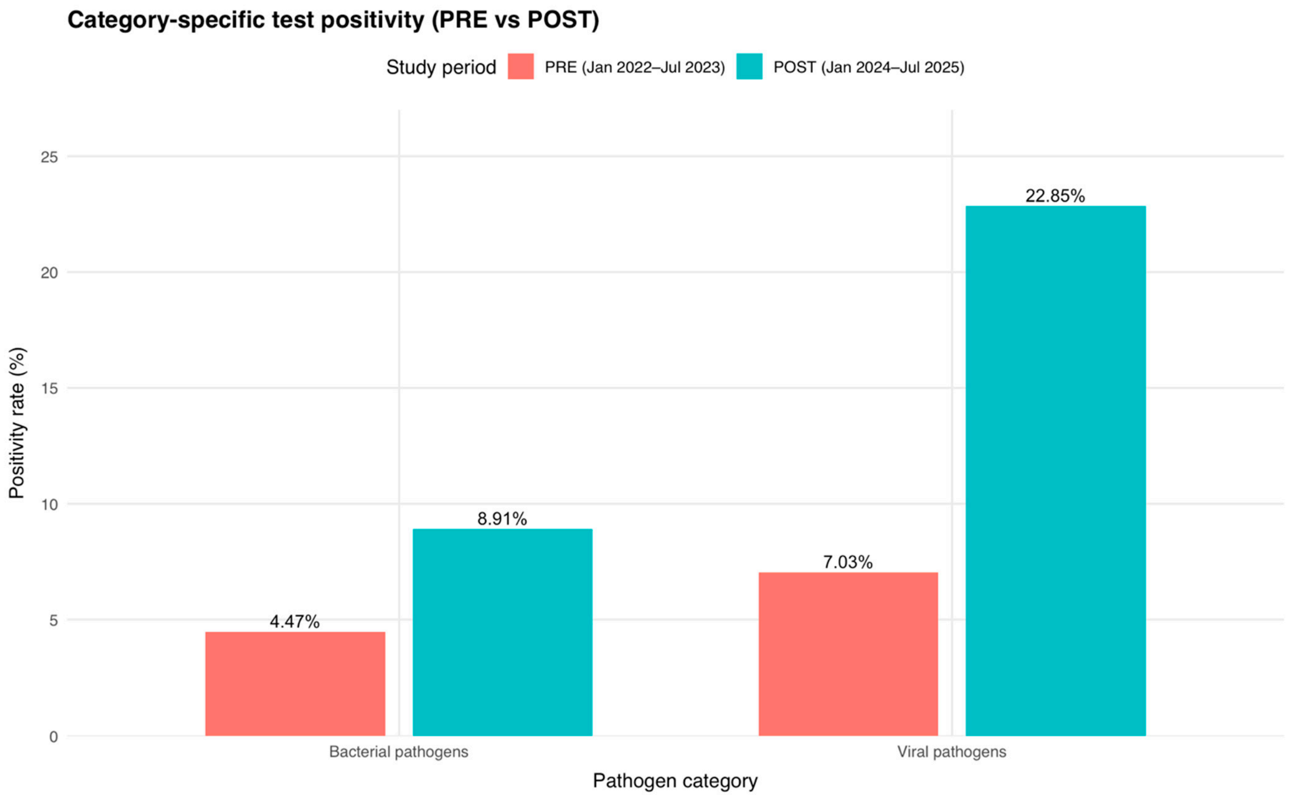

Figure S1. *Category-specific test positivity before (PRE) and after (POST) implementation of multiplex syndromic panels. Bacterial test positivity increased from 4.47% to 8.91%, and viral test positivity increased from 7.03% to 22.85% after implementation of syndromic testing (χ^2 test, $p < 0.001$ for both comparisons).*

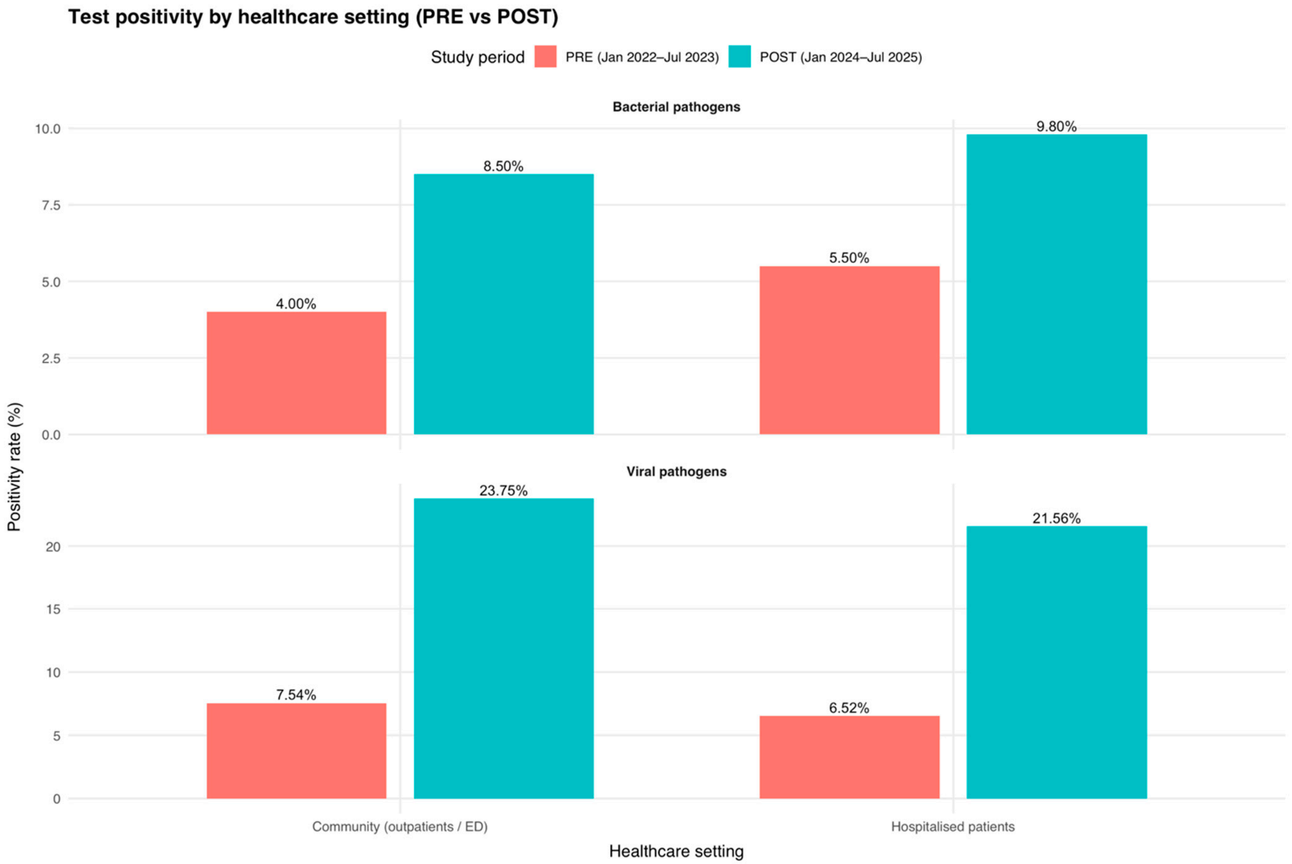

Figure S2. *Test positivity by healthcare setting before (PRE) and after (POST) implementation of multiplex syndromic panels. Bacterial test positivity increased in both community (4.0% to 8.5%) and hospitalised patients (5.5% to 9.8%). Viral test positivity also increased markedly in the community (7.54% to 23.75%) and hospital setting (6.52% to 21.56%) (χ^2 test, $p < 0.001$ for all comparisons).*

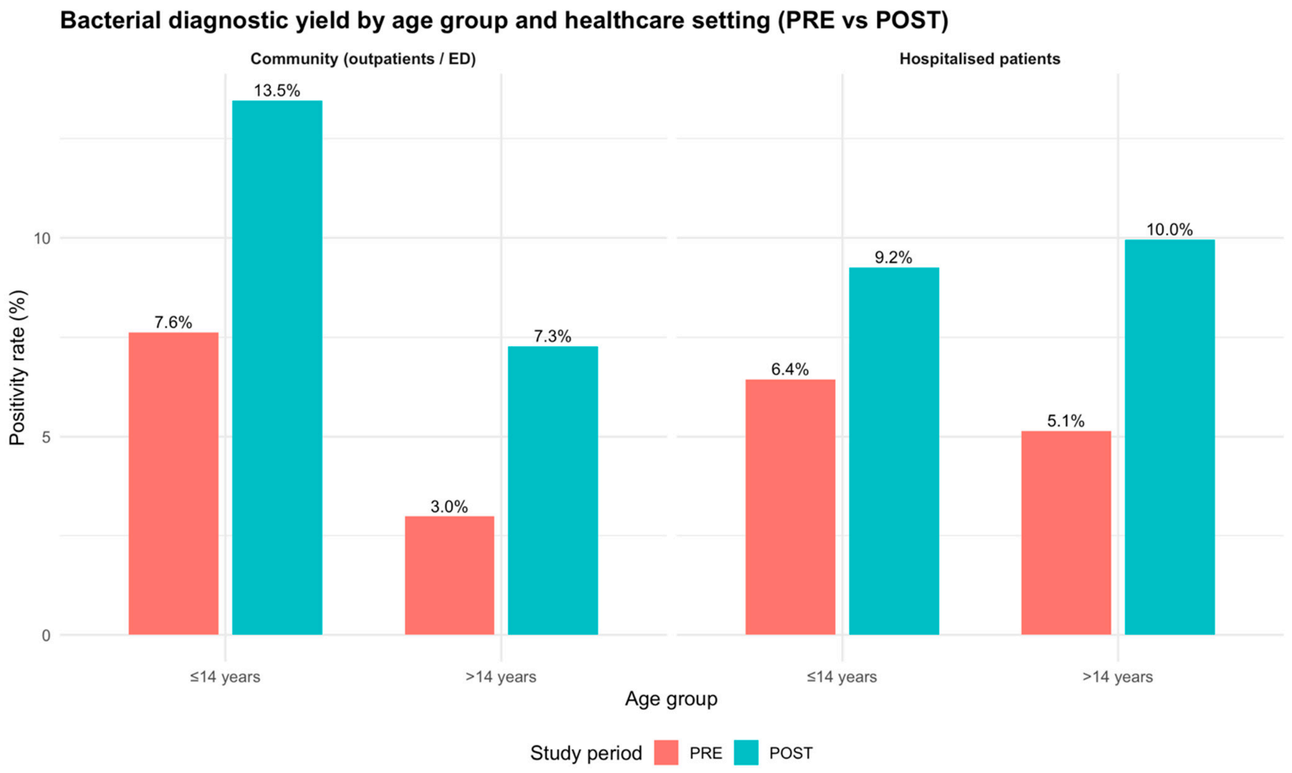

Figure S3. *Bacterial diagnostic yield by age group and healthcare setting before (PRE) and after (POST) implementation of multiplex syndromic panels. Bacterial test positivity increased significantly in both age groups across community (≤14 years: 7.6% to 13.5%; >14 years: 3.0% to 7.3%) and hospital settings (≤14 years: 6.4% to 9.2%; >14 years: 5.1% to 10.0%) (χ^2 test, $p < 0.001$ for all comparisons).*

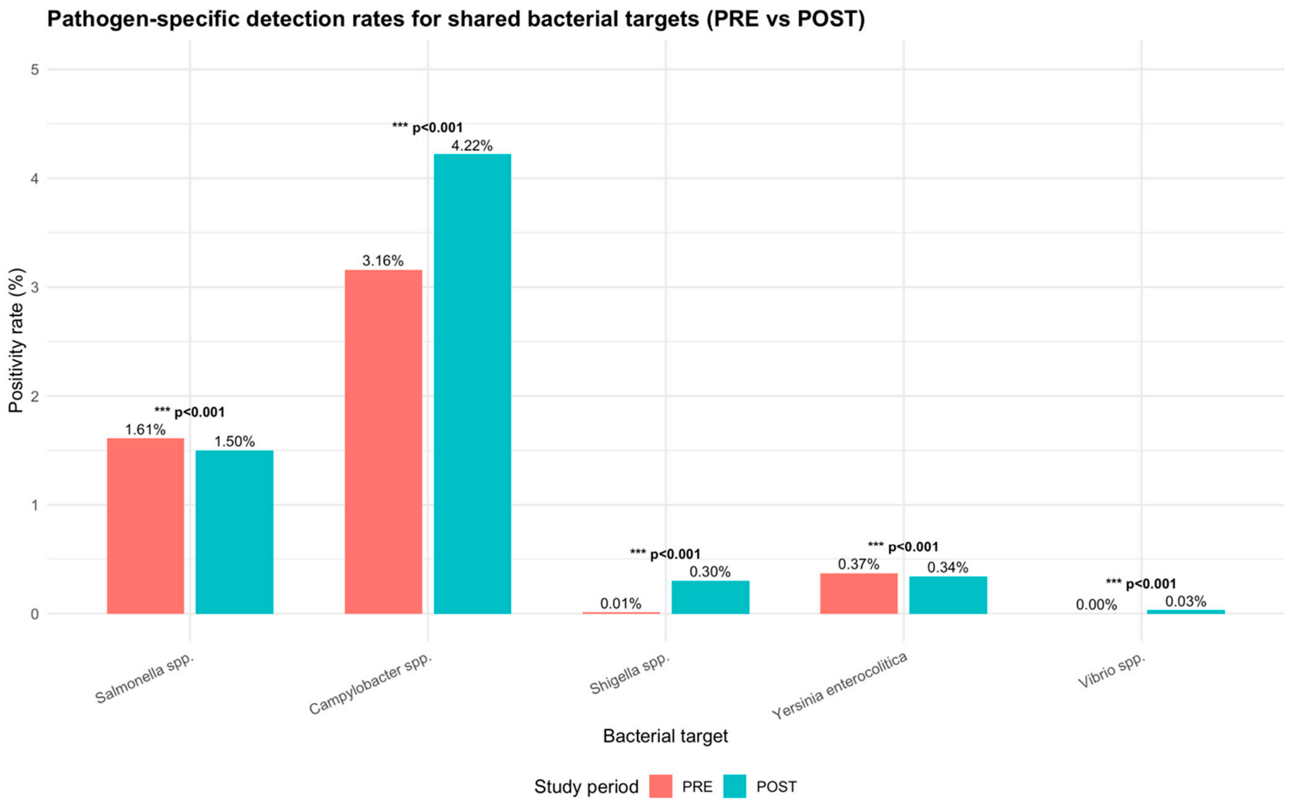

Figure S4. *Pathogen-specific detection rates for shared bacterial targets before (PRE) and after (POST) implementation of multiplex syndromic panels. Detection rates increased for Campylobacter spp. (3.16% to 4.22%) and Shigella spp. (0.01% to 0.30%), while remaining numerically stable for Salmonella spp. (1.61% to 1.50%) and Yersinia enterocolitica (0.37% to 0.34%). Vibrio spp. were detected only in the POST period (0.03%) (χ^2 or Fisher's exact test, $p < 0.001$ where applicable).*

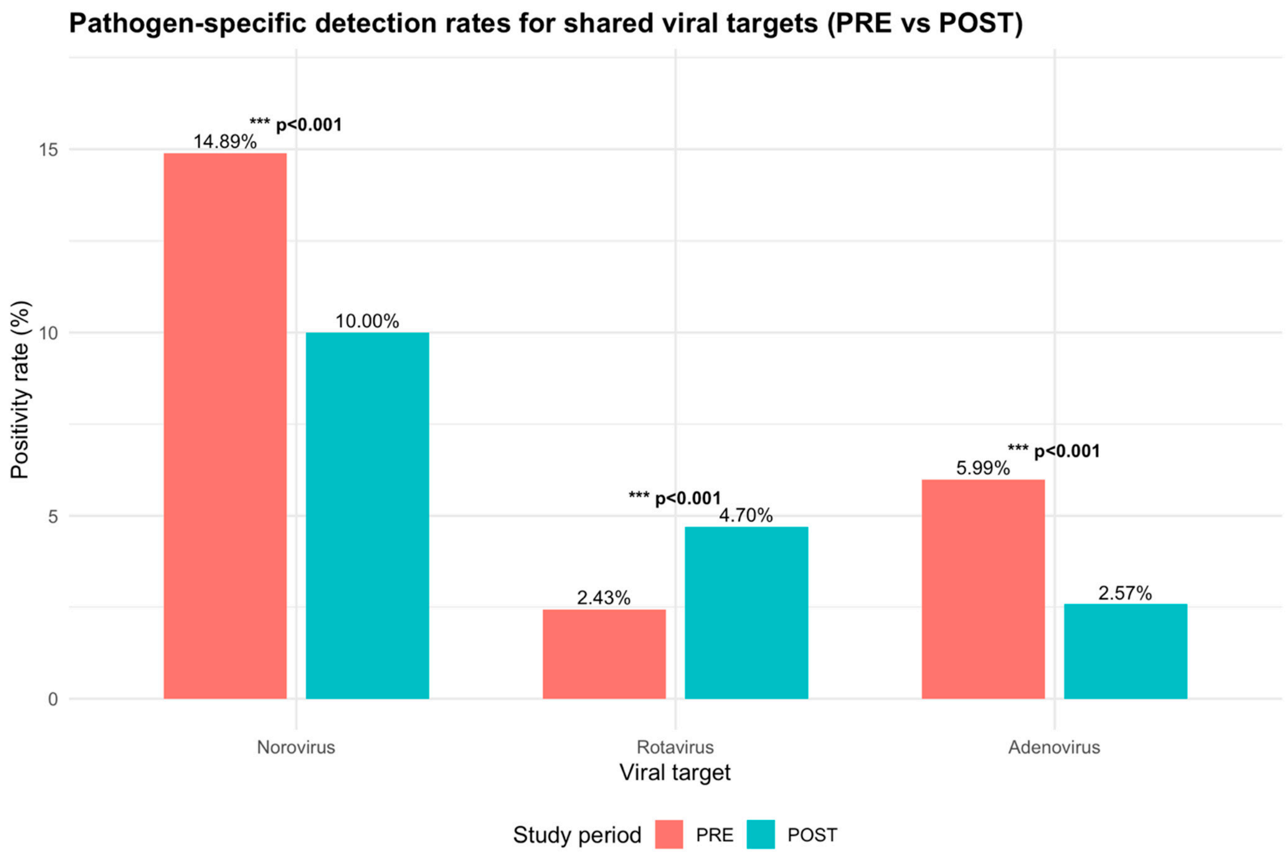

Figure S5. *Pathogen-specific detection rates for shared viral targets before (PRE) and after (POST) implementation of multiplex syndromic panels. Norovirus detection rate decreased from 14.89% to 10.00%, and Adenovirus from 5.99% to 2.57%, whereas Rotavirus increased from 2.43% to 4.70% after implementation (χ^2 test, $p < 0.001$ for all comparisons).*

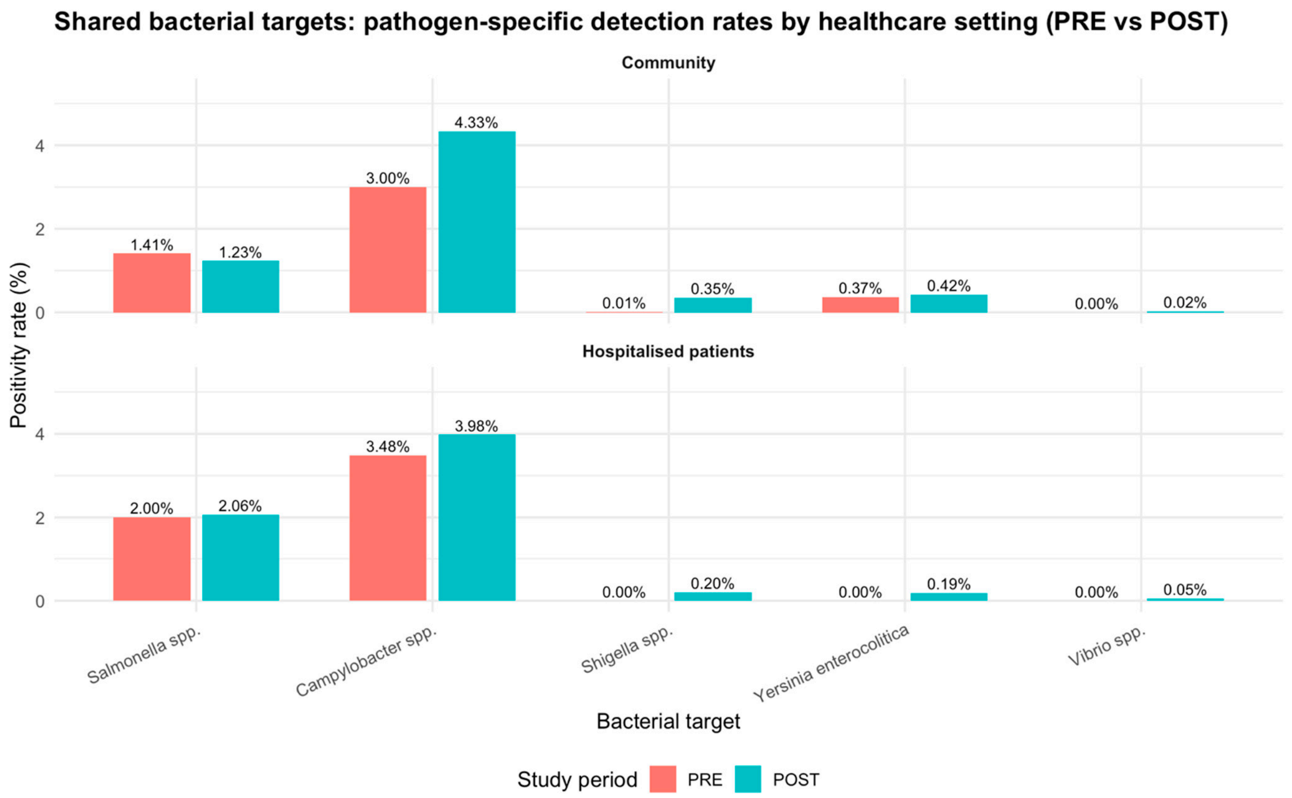

Figure S6.*Pathogen-specific detection rates for shared bacterial targets by healthcare setting before (PRE) and after (POST) implementation of multiplex syndromic panels. In the community setting, detection rates increased for Campylobacter spp. (3.00% to 4.33%) and Shigella spp. (0.01% to 0.35%), while remaining stable for Salmonella spp., Yersinia enterocolitica, and Vibrio spp. Among hospitalised patients, Shigella spp. increased (0.00% to 0.20%), whereas other targets showed no significant change (χ^2 or Fisher's exact test, $p < 0.001$ where applicable).*

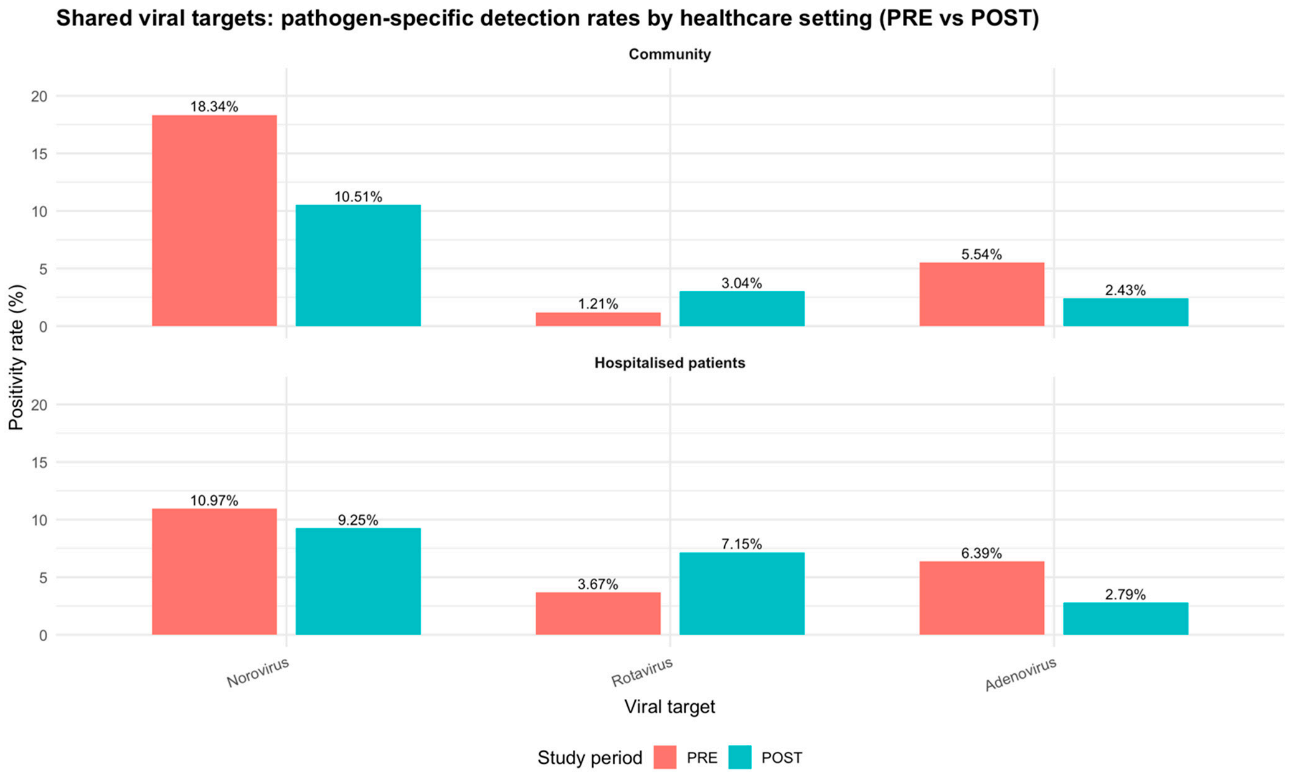

Figure S7.*Pathogen-specific detection rates for shared viral targets by healthcare setting before (PRE) and after (POST) implementation of multiplex syndromic panels. In the community setting, Norovirus and Adenovirus detection rates decreased (18.34% to 10.51% and 5.54% to 2.43%), while Rotavirus increased (1.21% to 3.04%) ($p < 0.001$). Among hospitalised patients, Rotavirus increased (3.67% to 7.15%) and Adenovirus decreased (6.39% to 2.79%) ($p < 0.001$), whereas Norovirus showed no significant change (10.97% to 9.25%; ns).*

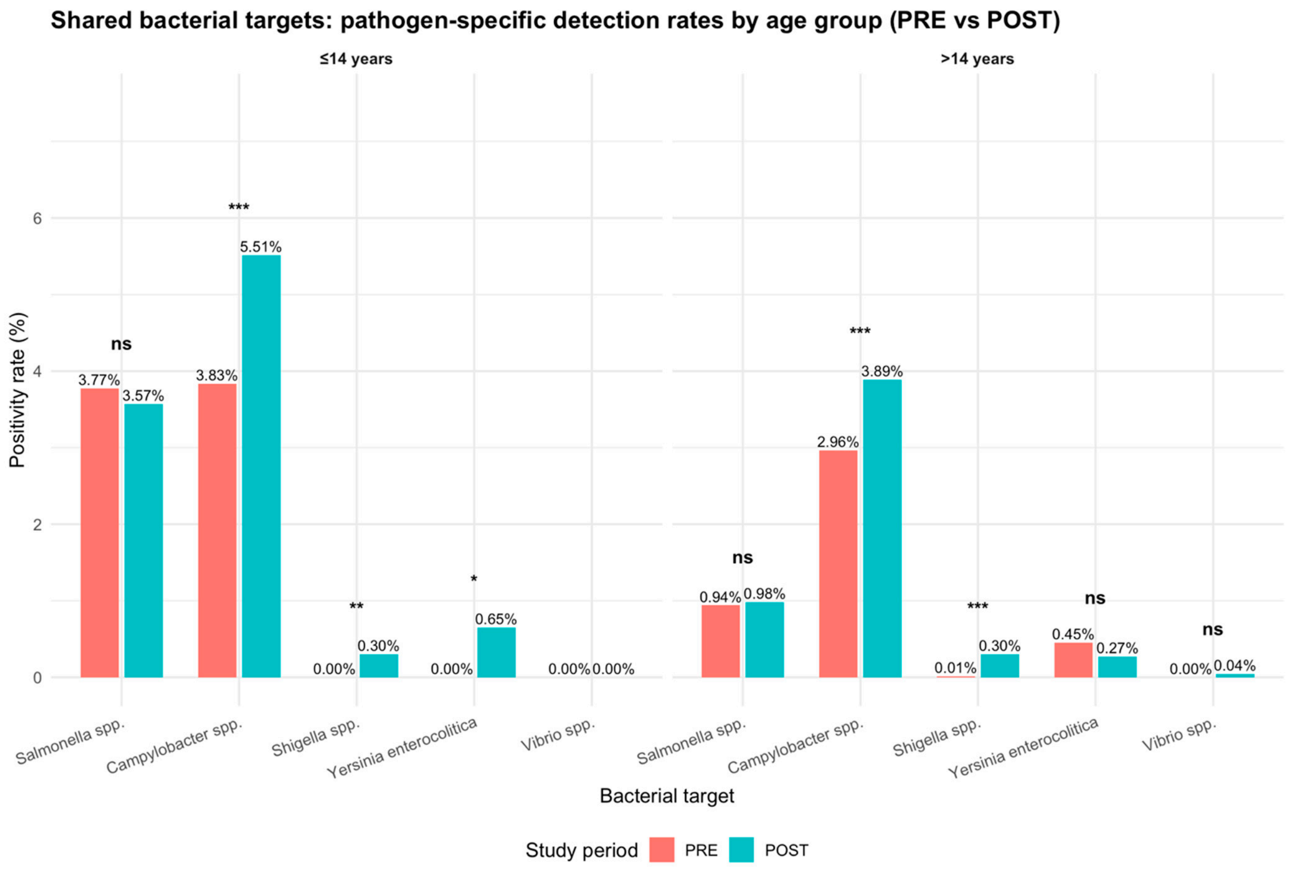

Figure S8. *Pathogen-specific detection rates for shared bacterial targets by age group before (PRE) and after (POST) implementation of multiplex syndromic panels. Among patients ≤14 years, detection rates increased for Campylobacter spp. (3.83% to 5.51%), Shigella spp. (0.00% to 0.30%), and Yersinia enterocolitica (0.00% to 0.65%), while Salmonella spp. remained stable. In patients >14 years, Campylobacter spp. (2.96% to 3.89%) and Shigella spp. (0.01% to 0.30%) increased, whereas Salmonella spp., Yersinia enterocolitica, and Vibrio spp. showed no significant change (*χ^2 *or Fisher's exact test,* $p < 0.05$ *where applicable).*

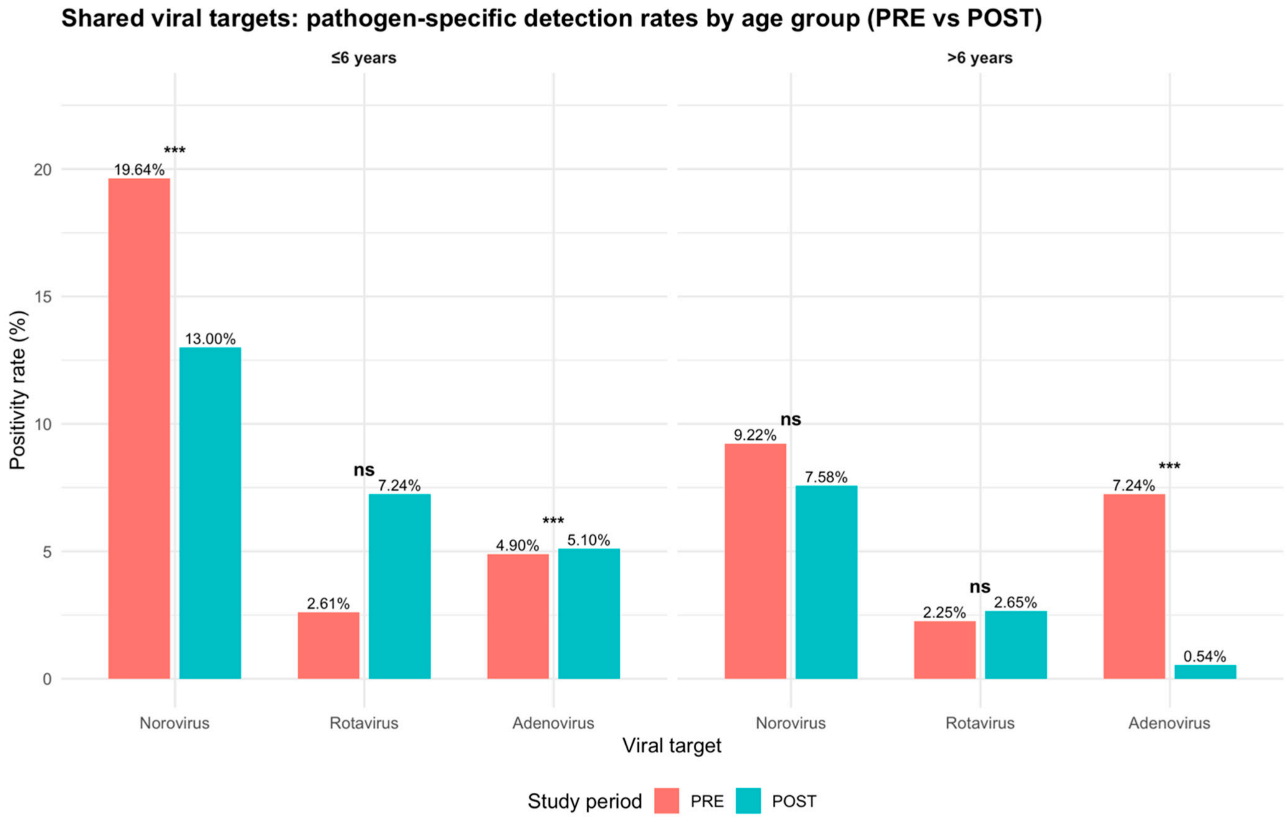

Figure S9. *Pathogen-specific detection rates for shared viral targets by age group before (PRE) and after (POST) implementation of multiplex syndromic panels. In children ≤6 years, Norovirus decreased (19.64% to 13.00%) and Rotavirus increased (2.61% to 7.24%), while Adenovirus remained stable (4.90% to 5.10%). In individuals >6 years, Adenovirus decreased markedly (7.24% to 0.54%), whereas Norovirus and Rotavirus showed no significant change (χ^2 test, $p < 0.001$ where applicable).*

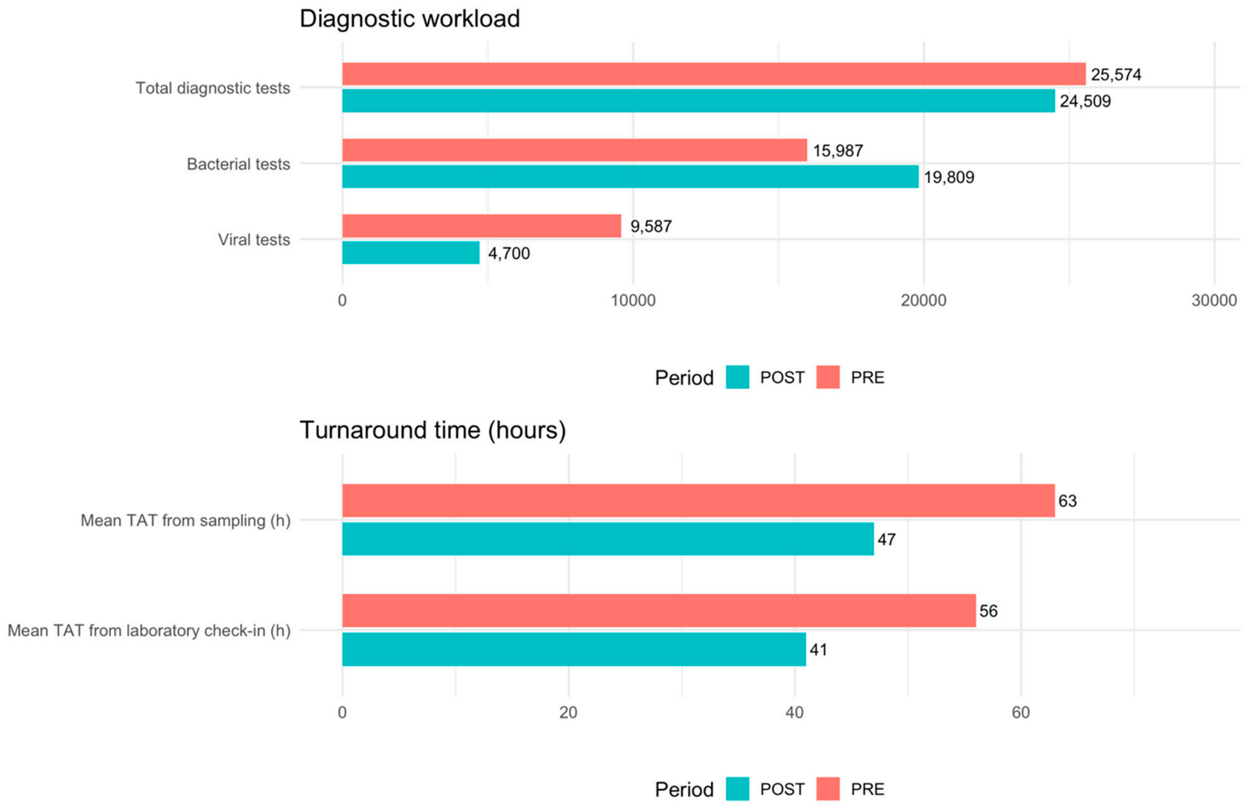

Figure S10. *Diagnostic workload and turnaround time before (PRE) and after (POST) implementation of multiplex syndromic panels.*
Total testing volume remained stable (25,574 vs 24,509; −4.2%), with increased bacterial testing (+23.9%) and reduced viral testing (−51.0%). Mean turnaround time decreased from 63 to 47 hours (sampling) and from 56 to 41 hours (laboratory check-in).
